# The impact of a direct to beneficiary mobile communication program on reproductive and child health outcomes: a randomised controlled trial in India

**DOI:** 10.1136/bmjgh-2022-008838

**Published:** 2022-07-14

**Authors:** Amnesty Elizabeth LeFevre, Neha Shah, Kerry Scott, Sara Chamberlain, Osama Ummer, Jean Juste Harrisson Bashingwa, Arpita Chakraborty, Anna Godfrey, Priyanka Dutt, Rajani Ved, Diwakar Mohan

**Affiliations:** 1 University of Cape Town, School of Public Health and Family Medicine, Cape Town, South Africa; 2 International Health, Johns Hopkins Bloomberg School of Public Health, Baltimore, Maryland, USA; 3 BBC Media Action, London, UK; 4 BBC Media Action, New Delhi, Delhi, India; 5 Oxford Policy Management, New Delhi, Delhi, India; 6 MRC/Wits-Agincourt Unit, University of the Witwatersrand, School of Public Health, Johannesburg, Gauteng, South Africa; 7 Computational Biology Division, Department of Integrative Biomedical Sciences, Institute of Infectious Disease and Molecular Medicine, University of Cape Town, Faculty of Health Sciences, Cape Town, South Africa; 8 Research and Evidence Practice, Oxford Policy Management, New Delhi, Delhi, India; 9 National Health Systems Resource Centre, New Delhi, Delhi, India; 10 Bill and Melinda Gates Foundation, Delhi, India

**Keywords:** child health, immunisation, nutrition, individual randomized trial

## Abstract

**Background:**

Direct-to-beneficiary communication mobile programmes are among the few examples of digital health programmes to have scaled widely in low-resource settings. Yet, evidence on their impact at scale is limited. This study aims to assess whether exposure to mobile health information calls during pregnancy and postpartum improved infant feeding and family planning practices.

**Methods:**

We conducted an individually randomised controlled trial in four districts of Madhya Pradesh, India. Study participants included Hindi speaking women 4–7 months pregnant (n=5095) with access to a mobile phone and their husbands (n=3842). Women were randomised to either an intervention group where they received up to 72 Kilkari messages or a control group where they received none. Intention-to-treat (ITT) and instrumental variable (IV) analyses are presented.

**Results:**

An average of 65% of the 2695 women randomised to receive Kilkari listened to ≥50% of the cumulative content of calls answered. Kilkari was not observed to have a significant impact on the primary outcome of exclusive breast feeding (ITT, relative risk (RR): 1.04, 95% CI 0.88 to 1.23, p=0.64; IV, RR: 1.10, 95% CI 0.67 to 1.81, p=0.71). Across study arms, Kilkari was associated with a 3.7% higher use of modern reversible contraceptives (RR: 1.12, 95% CI 1.03 to 1.21, p=0.007), and a 2.0% lower proportion of men or women sterilised since the birth of the child (RR: 0.85, 95% CI 0.74 to 0.97, p=0.016). Higher reversible method use was driven by increases in condom use and greatest among those women exposed to Kilkari with any male child (9.9% increase), in the poorest socioeconomic strata (15.8% increase), and in disadvantaged castes (12.0% increase). Immunisation at 10 weeks was higher among the children of Kilkari listeners (2.8% higher; RR: 1.03, 95% CI 1.00 to 1.06, p=0.048). Significant differences were not observed for other maternal, newborn and child health outcomes assessed.

**Conclusion:**

Study findings provide evidence to date on the effectiveness of the largest mobile health messaging programme in the world.

**Trial registration number:**

Trial registration clinicaltrials.gov; ID 90075552, NCT03576157.

**What is already known on this topic**

- Direct-to-beneficiary mobile communication programmes offer great potential to disseminate health information at a population level; reaching a large number of beneficiaries rapidly and at low cost. As a result, they are among the few examples of digital health solutions to scale widely in a range of settings globally. Despite their potential, no evidence exists of the impact of these programmes at scale in low-income and middle-income countries where the majority of maternal and child deaths occur each year. The evidence that does exist is limited to smaller scale pilots; key programme components of which often change in the process of scaling up.

**What this study adds**

- Kilkari is the largest direct-to-beneficiary mobile communication programme in the world and has reached over 10 million women and their families across 13 states in India. Our study is the first randomised controlled trial conducted to date of a beneficiary mobile communication programme at scale. Exposure to Kilkari was significantly associated with improvements in a few important health practices, including the use of reversible contraceptive methods, but not others, including exclusive breast feeding. Subgroup analyses highlight the differential impact among key population segments, including the poorest.

**How this study might affect research, practice or policy**

- Study findings provide the most conclusive evidence to date on the effectiveness of the largest beneficiary mobile communication programme in the world. The differential targeting of key population segments could serve to deepen the impact. Further research is needed to assess the impact of shorter duration campaigns less susceptible to SIM change, network disruptions and population migration.

## Background

Direct-to-beneficiary mobile health (mHealth) programmes which provide stage-based health information to new and expectant mothers have proliferated rapidly throughout the last decade.^1–3^ In low-income and middle-income countries where the majority of maternal and child deaths occur, mHealth messaging programmes are among the few examples of digital health programmes to successfully scale. Five programmes globally have scaled to reach over a million subscribers in Bangladesh,^4 5^ India,^6 7^ South Africa^1^ and Tanzania.

Evidence linking scaled mHealth messaging programmes to changes in health outcomes is limited^7–12^; a factor which may impede sustainability and further expansion.^13^ The scant evidence available suggests varied impacts on a range of outcomes including improvements in birth weight^7^; infant feeding practices^7 8^; utilisation of early and complete antenatal care (ANC),^8 9^ facility-based skilled birth attendance,^10 12^ maternal and childhood immunisation services^7^; and reductions in perinatal mortality.^11^ However, efforts to compare and generalise these findings have been hampered by wide variations in study contexts and programme components including the content, modality, frequency of messages and supporting programmatic activities.^14^ The small scale of these programmes at the time that they were evaluated also limits the generalisability of the findings observed. As programmes scale they often undergo significant changes in design, implementation and resource availability, which can lead to a ‘voltage drop’ in a range of factors including reach, exposure and impact on beneficiary practices and health behaviours.^2 15^ These challenges underscore the need for evidence at each stage of an mHealth programme’s maturity from pilot to delivery of services at scale, to a large number of beneficiaries.

Kilkari is the world’s largest mHealth messaging programme of its kind. Kilkari is an outbound service that makes weekly, stage-based, prerecorded calls about reproductive, maternal, neonatal and child health (RMNCH) directly to families’ mobile phones, starting from the second trimester of pregnancy until the child is 1 year old. BBC Media Action designed and piloted Kilkari in the Indian state of Bihar in 2012–2013, and then redesigned and scaled it in collaboration with the Ministry of Health and Family Welfare (MOHFW) between 2015 and 2019. Prior to its transition to MoHFW in April 2019, Kilkari had been scaled to 13 states across India and reached over 10 million subscribers.^16^

From 2018 to early 2020, an individually randomised controlled trial (RCT) was conducted in four districts of Madhya Pradesh to determine the effectiveness of Kilkari. This study sought to assess whether exposure to mHealth information calls during pregnancy and postpartum improved infant feeding and family planning practices. This is the first study to evaluate the impact of a maternal mHealth messaging programme at scale; additional details on the evaluation protocol have been published elsewhere.^17^

## Methods

### Trial design

The study is an individually RCT with a parallel and unblinded design.

### Participants

At the time of randomisation in late 2018, women enrolled to the study (n=5095) were 12–34 weeks of gestation, more than 18 years of age, could speak and understand Hindi, and owned or had access to a mobile phone during the day when Kilkari calls were likely to come. Women were excluded who did not consent or who were mobile subscribers of Bharat Sanchar Nigam Limited (the state-owned telecommunications company) due to poor network coverage in the RCT districts in Madhya Pradesh. Given the shared nature of mobile phones, the husbands of women enrolled to the study were additionally interviewed as part of endline survey activities.

### Study setting

Women in India have limited access to and use of mobile phones. Despite near universal household level phone ownership (92.8%), only 47.8% of Indian women have access to a mobile phone (41.6% in rural areas and 62.7% in urban).^18^ In the central Indian state of Madhya Pradesh (population 75 million) which is home to an estimated 20% of India’s total population, 19.1% of women rural areas and 49.5% in urban had access to a phone that they themselves could use in 2015.^19^ Beyond limitations in women’s phone access, Madhya Pradesh’s population health status falls below national level averages for most RMNCH indicators. In 2015, only 49.6% of women reported using any modern method for family planning and 12.1% reported having an unmet need for family planning.^19^ While over half (53.0%) of pregnant women attended ANC clinics in the first trimester, only 35.7% received the recommended four ANC visits.^19^ Despite high rates of institutional delivery (80.8%), only 54.9% of women and newborns received a postnatal health check within 2 days following birth.^19^ Among children, 34.4% were breastfed within 1 hour of birth and 58.2% were exclusively breastfed until 6 months of age.^19^ One in four children (25.8%) were wasted (weight-for-height) and 42.0% were stunted (height-for-age).^19^ Among children 12–23 months of age, 53.6% were fully immunised (Bacille Calmette-Guerin (BCG), measles and three doses each of polio and Diphtheria, Pertussis, and Tetanus (DPT)).^19^

### Intervention description

**Figure 1:**
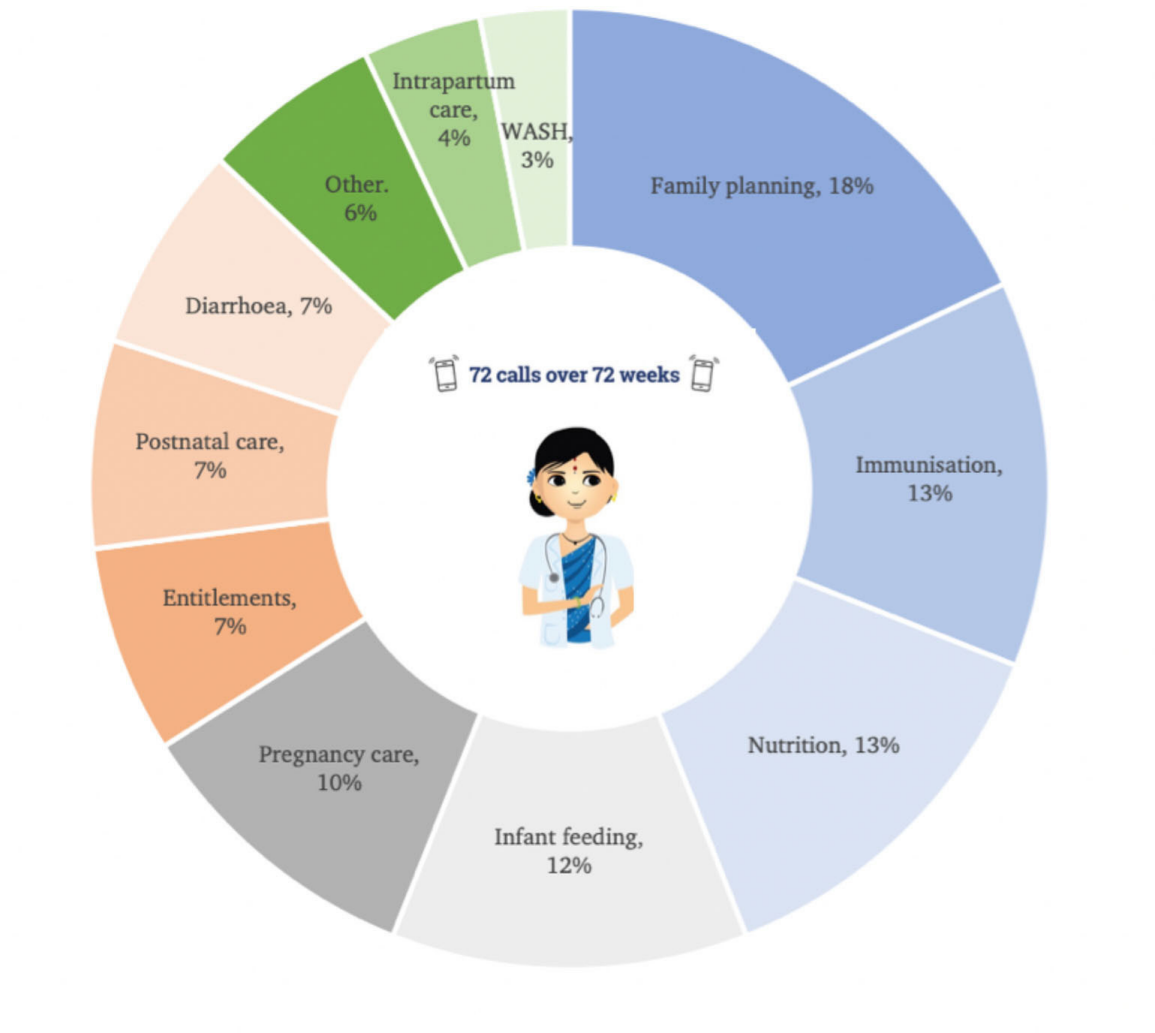
Summary of Kilkari content during pregnancy and up to 1 year post partum. WASH, water and sanitation.

Kilkari is comprised of 90 min of RMNCH content sent via 72 once weekly voice calls: 24 during pregnancy, 24 within the first 6 months postpartum and 24 from 7 to 12 months postpartum. Individual calls span an average of 77 s in duration and are framed as coming from ‘Dr Anita’. Across health content areas, 18% of cumulative call content is on family planning (benefits of family planning, modern reversible methods, sterilisation, pregnancy tests); 13% on child immunisations (diseases covered, doses); 13% on nutrition (malnutrition, growth monitoring, anaemia); 12% on infant feeding (quality of food, breast feeding, complementary feeding, anaemia); 10% on pregnancy care (ANC, institutional delivery, rest, nutrition, tetanus toxoid, emergency services); 7% on entitlements; 7% on diarrhoea; 7% on postnatal care (newborn danger signs, cord care, hypothermia); and the remainder on a range of topics including intrapartum care, water and sanitation (WASH) and early childhood development (figure 1).

The mobile numbers provided by women randomised to receive Kilkari began receiving calls no later than 8 months (34 weeks) after conception, making them eligible for at least 57 of the 72 messages. The timing of RCT enrolment was intended to mirror the timing of enrolment to Kilkari elsewhere in India. Once subscribed, Kilkari used an algorithm to retry mobile numbers up to nine times—three times in each week—three times on the first day, and twice then twice each day for the next 3 days—to reach a subscriber. Respondents were given the option to unsubscribe from the service at any time.

### Outcomes

The primary outcome of this study was the reported practice of exclusive breast feeding for infants 0–6 months of age. The secondary outcome was the use of modern reversible contraceptive methods (including intrauterine contraceptive device (IUCD), injectables, oral contraceptive pills, emergency contraceptive pills, condoms) at 1 year post partum. The latter outcome was added after the trial registration but included in the published protocol. Three reasons underpin its inclusion. First, family planning messages constitute the largest overall message share (figure 1). Second, family planning calls occur throughout the extended postpartum window allowing for a large window of exposure to the intended messages. Third, the complexity of phone sharing practices in this population means that men may be the actual listeners of some of the Kilkari calls.^20^ Family planning represents includes behaviours which depend on both men and women both of whom may have been exposed to Kilkari calls. The outcome of immediate/early breast feeding noted in the trial registration was not emphasised as a primary or secondary outcome in our protocol. The study team felt that changes in these outcomes might be challenging to observe for two reasons. First, immediate/early breast feeding can be supply side dependent in the case of facility based and/or skilled attendant deliveries (which is why we reported it for normal deliveries and all deliveries) and thus, information provided to pregnant women is unlikely to move the indicator. Second, there are only two Kilkari messages (message 17: pregnancy month 8, week 1 and message 23: pregnancy month 9, week 3) which include content on early and immediate breast feeding.

To assess the impact of Kilkari exposure on RMNCH outcomes, endline surveys were administered to enrolled women and their husbands after 12 months postpartum. The women’s endline survey included modules on RMNCH knowledge, practice, decision-making and demand for information and supply side services for the following health areas: pregnancy and delivery care, newborn care, child health, infant and young child feeding (IYCF), family planning, media consumption and reported demand for and receipt of health services from frontline health workers. The men’s survey sought to measure RMNCH knowledge; mobile phone ownership, use and literacy; and women’s access to phones. In addition to relying on respondent recall to answer survey questions, investigators also collected data from the participant’s Mother and Child Health card, which is issued to every pregnant woman at the local health facility and updated each visit and includes the child’s immunisation record.

### Sample size

To detect a 5% difference in the reported practices of exclusive breast feeding and reversible modern contraceptive use assuming an alpha of 0.05, 80% power, the estimated sample size for a two-sample proportions test would be 3200 women. After adjusting for a design effect (variance inflation factor) of 1.25 due to clustering at the level of the Community Development Block (India’s subdistrict level administrative units), a 20% loss to follow-up from enrolment to endline, and a potential loss of 35% of women due to poor reporting of phone numbers and/or changes to original phone numbers provided, a total of 5100 women were targeted for enrolment in the study. We assumed that 40% of women in the reproductive age would have access to a mobile phone in 2018 at the start of the study, and that 12.5% of households would have a pregnant woman between 4 and 7 months, and thus anticipated needing to visit up to 750 000 households across the four study districts.

### Enrolment of participants

Study participants were identified through a household listing survey carried out from July to October 2018 which included an intensive mapping of households and their residents and screened participants for eligibility. Women identified during the listing as meeting the eligibility criteria (Hindi speaking, 4–7 months pregnant, with access to a mobile phone during the day) were screened again by a separate baseline survey team. Those confirmed to meet eligibility criteria were administered a 1.5 hour baseline survey inclusive of modules on sociodemographic characteristics; phone ownership, use and digital competency; RMNCH knowledge and practices. Following the baseline survey, study participants who completed the face to face survey and consented to receive Kilkari messages were randomised to receive Kilkari calls (intervention) or not receive messages (control). Exposure to Kilkari spanned through the child’s first birthday. Study participants were interviewed at 12 months post partum as part of endline survey activities carried out from November 2020 to March 2021. No harms are anticipated for study participants enrolled to either study arm.

### Randomisation and masking

The participants were randomised after stratification on a range of variables potentially associated with exposure (listening to messages) and likely to influence outcomes, including gestational age, parity, age of woman and ownership of phone. Stratification sought to ensure a balance of covariates between the intervention and control groups. The individual randomisation procedure was done on a block-by-block basis (block randomisation) after enrolment was completed for each block. This ensured similar number of intervention and control subjects for each community development block. Randomisation was performed by picking a random sample of participants for each Block using the *sample* command in Stata with the use of the above listed variables as stratifiers. The randomisation and allocation processes were carried out by the study investigators in late 2018 following the completion of baseline survey activities. The random sample picked was allocated to receive the intervention immediately after randomisation. The participants could not be blinded due to the nature of the intervention. The data collectors administering surveys were not aware of the allocation.

### Statistical methods

To assess exposure to Kilkari content, call data records from the interactive voice response (IVR) system were linked to baseline and endline survey data. Listening patterns were assessed for each subscriber by call, for the duration of the their subscription to Kilkari. The content of the Kilkari calls was mapped to key health outcomes including knowledge and practice. Exposure was defined at a listening threshold of 50% or more of the cumulative duration of the calls mapped to the outcome. For example, 7 Kilkari calls (week 20, 24, 29, 34, 65, 66, 68) included content on reversible modern contraceptive methods, for a total of 10.5 min of audio content on this topic (online supplemental table 1). To be ‘exposed’ subscribers would have had to listen to 50% or more of the cumulative message total of these messages, for example, 5 min or more of the contents.

10.1136/bmjgh-2022-008838.supp1 Supplementary data

Primary analyses of outcomes were done with modified intention-to-treat (ITT) analyses at the individual level so that outcomes were analysed regardless of the degree of listening to Kilkari. The term modified refers to the requirement of being able to determine the outcomes beyond 1 year of the postpartum period (ie, only those who were reached by the endline survey were included in the analysis). Relative risk (RR) for primary and secondary outcomes was calculated for the intervention group compared with the control group using log-binomial regression with general estimating equations to account for correlation within villages.

To assess the impact of exposure on outcomes, compliance adjusted treatment effects were additionally generated using the instrumental variable (IV) methodology. The study arm based on randomisation was considered the instrument variable. As noted above, exposure was defined as listening to at least 50% of the cumulative duration of the calls mapped to the outcome. RR estimates were calculated using log binomial models for each outcome with adjustment for clustering at the village level. The IV analysis was carried out using the IVREG package in R.^21^ Frequencies and proportions are used to describe differences across intervention and control groups (ITT) as well as those exposed to 50% or more of Kilkari content.

### Role of the funding source

The Bill and Melinda Gates Foundation had no role in the study design; collection, analysis and interpretation of data; in the writing of the report; or in the decision to submit the paper for publication. All authors confirm that they had full access to all the data in the study and accept responsibility for the publication submitted.

### Patient and public involvement

Patients were first engaged on identification in their households as part of a household listing carried out in mid/late 2018. Those meeting eligibility criteria were interviewed as part of the baseline survey, and ultimately randomised to the intervention and control arms. Prior to the administration of the baseline, a small number of patients were involved in the refinement of survey tools through qualitative interviews, including cognitive interviews, which were carried out to optimise survey questions, including the language and translation used. Finalised tools were administered to patients at baseline and endline, and for a subsample of the study population, additional interviews carried out over the phone and via qualitative interviews between the baseline and endline surveys. Unfortunately because of COVID-19 and associated travel restrictions, patients could not be involved in the dissemination of study findings. However, public dissemination of the results has occurred through a number of global level presentations.

## Results

### Sample characteristics

**Figure 2:**
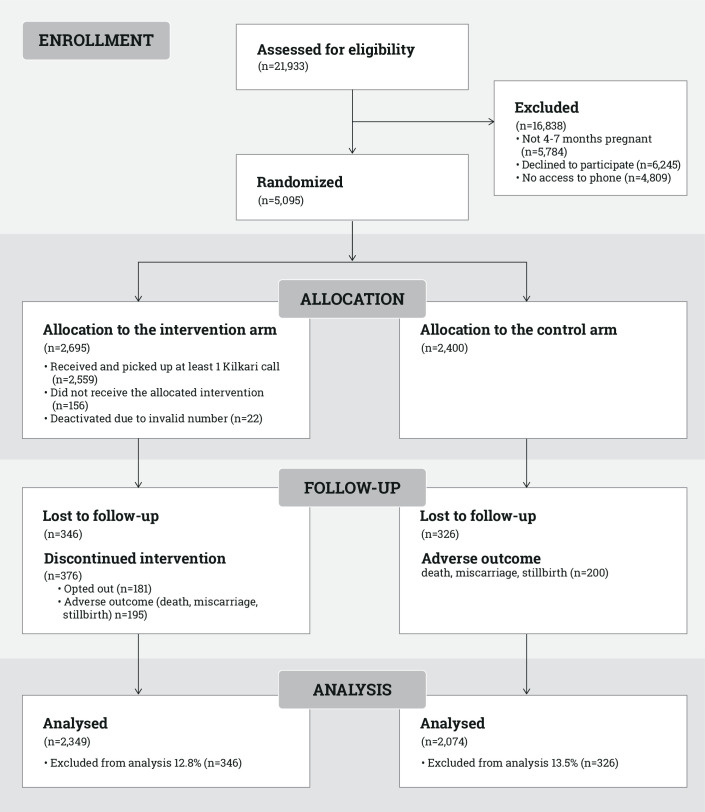
Consolidated Standards of Reporting Trials flow diagram.

Among the 5095 women enrolled at baseline, 2695 were randomised to the intervention arm and 2400 to the control arm (figure 2). An estimated 95% (n=2559) of the mobile numbers provided by women randomised to the intervention arm received at least 1 Kilkari call that was answered. Table 1 shows the characteristics for the ITT population of women (n=5095) enrolled at baseline and their husbands (n=3842) interviewed at endline.

**Table 1:** Sample characteristics for women and men’s surveys in four district of Madhya Pradesh

	Women’s baseline survey (n=5095)				Men’s survey (n=3842)			
	Intervention		Control		Intervention		Control	
	n	%	n	%	n	%	n	%
Overall	2695	53	2400	47	2033	53	1809	47
Gestational age at time of enrolment								
<20 weeks	575	21	515	21	447	22	392	22
20–28 weeks	1352	50	1205	50	1011	50	898	50
28–34 weeks	768	28	680	28	575	28	519	29
Age at time of enrolment in years								
18–24	1553	58	1422	59	322	16	314	17
25–34	1070	40	953	40	1435	71	1269	70
35+	72	3	25	1	276	14	226	12
Literacy: able to read sentence	1507	56	1373	57	1502	74	1377	76
Education								
No schooling	311	12	249	10	86	4	71	4
Primary school completed	457	17	407	17	346	17	290	16
Secondary school completed	1700	63	1526	64	1308	64	1160	64
Greater than secondary school	227	8	218	9	293	14	288	16
Employed	1038	39	883	37	2020	99	1796	99
Socioeconomic status								
Poorest	542	20	477	20	345	17	279	15
Poorer	564	21	455	19	396	19	316	17
Middle	514	19	505	21	391	19	392	22
Richer	530	20	489	20	431	21	400	22
Richest	545	20	474	20	470	23	422	23
District								
Rewa	1523	57	1397	58	970	48	880	49
Mandsaur	430	16	391	16	382	19	361	20
Rajgarh	510	19	438	18	473	23	407	22
Hoshangabad	232	9	174	7	208	10	161	9
Parity: had previous child	1587	59	1414	59	1198	59	1064	59
Ethnicity/caste								
General caste	595	22	538	22	398	20	363	20
OBC	1258	47	1128	47	982	48	932	52
Scheduled caste	540	20	461	19	441	22	322	18
Scheduled tribe	302	11	273	11	212	10	192	11
Any male children	1678	62	1536	64	–	–	–	–
Baseline ever use modern contraceptive methods	634	24	627	26	–	–	–	–
Phone ownership, characteristics								
Own phone	2014	75	1846	77	1924	95	1718	95
Type of phone: smart phone	591	22	547	23	1041	51	968	54
Shared phone*	1317	49	1097	46	1636	80	1473	81
Phone functions†	1480	55	1285	54	1446	71	1274	70
Mean hours per day phone in possession	18	18	18	18	20	20	20	20
Credit on phone (mean)	11	11	11	11	–	–	–	–
Digital use								
Store contacts on phone	2197	82	1980	83	1734	85	1542	85
Able to navigate IVR prompts	2288	85	2056	86	1939	95	1713	95
Able to open and read SMS	801	30	728	30	1231	61	1143	63
Give a missed call	1900	71	1687	70	1685	83	1487	82

*Includes phone sharing among phone owners.

†Phone holds charge, screen not cracked, key pad functional.

OBC, other backward castes.

### Exposure to Kilkari calls

**Figure 3:**
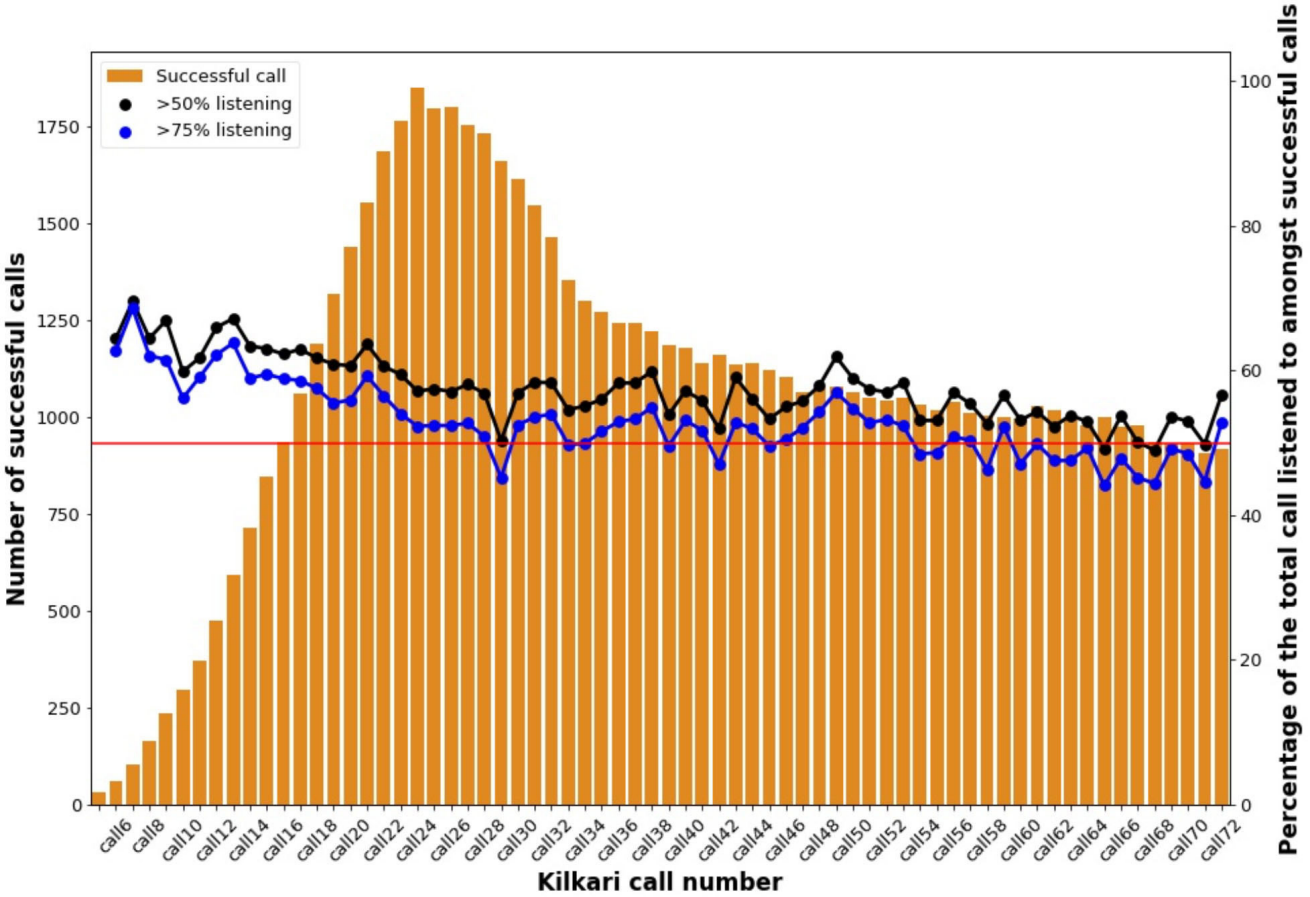
The number of successful* Kilkari calls and per cent of listening by call over time for those randomised to the intervention arm in four districts of Madhya Pradesh, India. *Successful calls are those which are delivered to the handset and answered for 1 s or more.

The mobile numbers provided by those randomised to the intervention arm began receiving Kilkari calls no later than the first week of the eighth month of their pregnancy (Kilkari weekly call 17). Successful calls are those that reach the handset and are answered. We cannot identify who answered the call. Figure 3 depicts the number and duration of successful calls for each of the Kilkari calls among the mobile numbers provided by those randomised to the intervention arm. As denoted by the orange bars, the number of successful calls peaked at Kilkari call 22 (pregnancy month 9, week 2) and declined gradually from call 25 (baby month 1, week 1) to 38 (baby month 4, week 2). Declines in successful calls were attributed primarily to deactivations as a result of adverse events (miscarriage, death, stillbirths), SIM change and increases in call non-delivery rates. From the timing of the baseline survey to 12 months postpartum when the endline survey occurred, 44% of women enrolled into RCT changed their SIM cards and thus were no longer receiving Kilkari calls. Women who retained the same SIM card from baseline to endline tended to be more educated, within the higher socioeconomic strata, and advantaged caste groups. Call non-delivery rates increased throughout 2018 and were underpinned by the bankruptcy of Reliance communications, one of India’s leading mobile network providers.

High listenership, depicted by the black and blue horizontal lines in figure 3, is defined as the proportion of subscribers who listened to at least 50% and 75%, respectively, of the total content of an individual call. Across individual Kilkari calls, high listenership rates were stable over time, ranging from 45% to 65% among successful calls. An average of 65% of subscribers listened to more than 50% of the cumulative total of successful calls while 31% listened to more than 75% of the total content of calls. The odds of listening to 50% or more of the cumulative content were greater among those enrolled earlier in pregnancy, with the same phone number from baseline to endline surveys, and with double digit numeracy (online supplemental figure 1).

**Figure 4:**
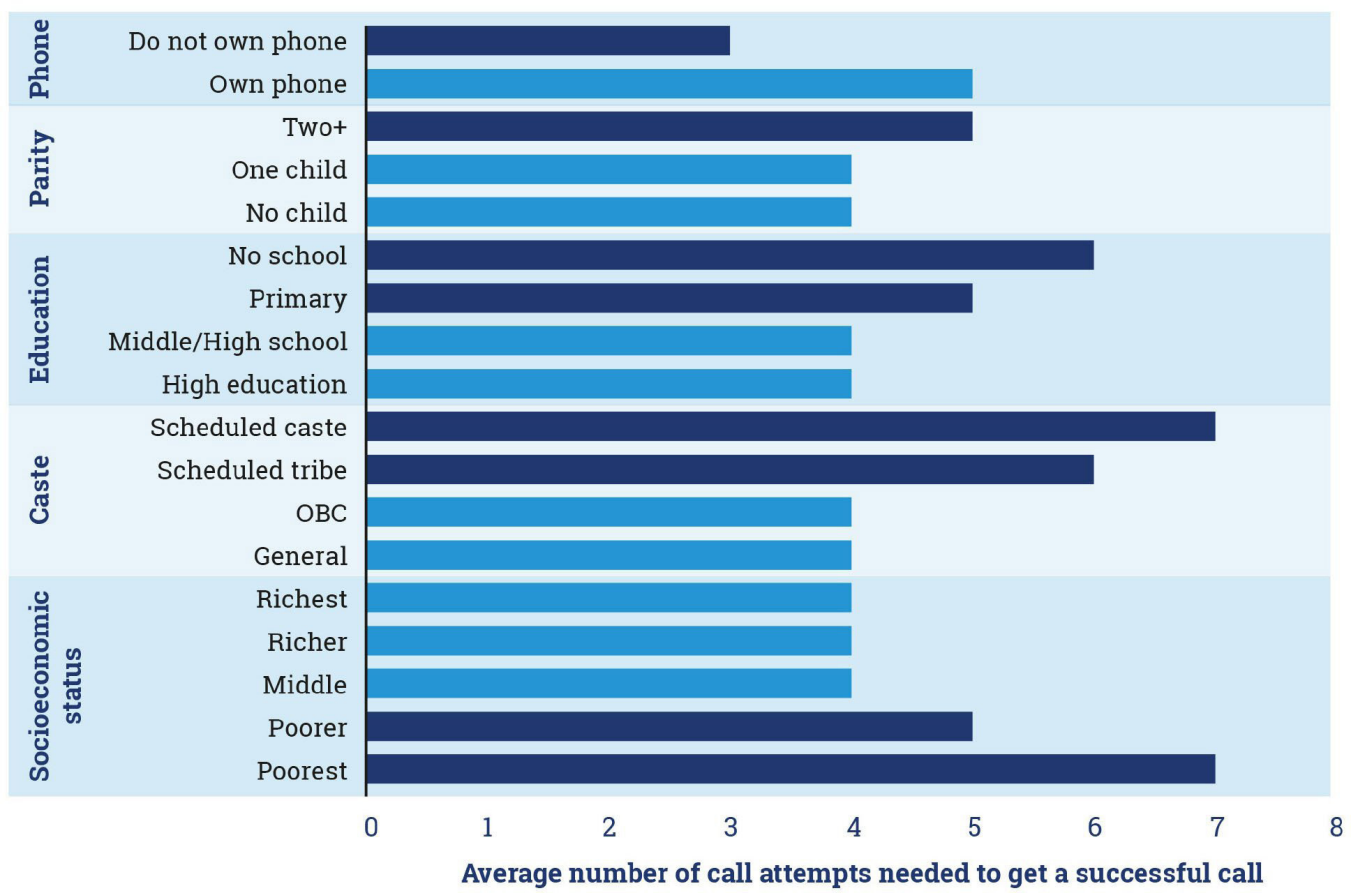
Number of call attempts needed to reach subscribers by sociodemographic characteristic. Dark blue bars denote the more marginalised subscribers as compared with those in the lighter blue. OBC, other backward castes.

The Kilkari programme’s algorithm that keeps calling subscribers who do not answer up to nine times each week increased the proportion of successful calls, especially among subscribers in the most marginalised demographic groups (figure 4). On average, seven call attempts were needed to reach subscribers in the poorest socioeconomic strata as compared with four attempts needed to reach those in the higher (middle, richer and richest) socioeconomic strata. Across ethnic groups, seven call attempts were needed on average to reach scheduled caste subscribers versus four in the more advantaged other backward castes and general castes. Similar patterns were observed for those without education and with two or more children. Efforts to assess the timing of calls per day are reported elsewhere^22^ and broadly suggest that call answer rates were lowest earlier in the day and overall for those in the most marginalised sociodemographic groups.

### Primary outcome: exclusive breast feeding

Kilkari was not observed to have a significant impact on exclusive breast feeding (ITT, RR: 1.04, 95% CI 0.88 to 1.23, p=0.64; IV, RR: 1.10, 95% CI 0.67 to 1.81, p=0.71). However, the proportion of children born through a normal delivery who were immediately breastfed was 3.0% higher in the control arm as compared with the intervention (ITT, RR: 0.96, 95% CI 0.93 to 1.00, p=0.027). Significant differences were not observed for other IYCF outcomes assessed, including complementary feeding and the provision of iron folate (table 2).

**Table 2:** Infant and young child feeding and family planning practices in four districts of Madhya Pradesh

Description	Women’s endline survey (n=4423)											
	Prevalence across study arms			Intention to treat			Prevalence across exposed versus not exposed groups			Instrumental variable		
	Int.	Comp.	Difference	RRs	95% CI	P value	Exposed	Not exposed	Difference	CATE	95% CI	P value
Family planning method use												
Reversible method use since birth of child	33.7	30.0	3.68	1.12	1.03 to 1.21	0.007	38.5	31.1	7.40	1.72	1.06 to 2.77	0.028
*Condom*	*26.7*	*23.8*	*2.87*	*1.12*	*1.01 to 1.23*	*0.024*	*30.6*	*24.6*	6.00	*1.76*	*0.94 to 3.31*	*0.077*
Contraceptive pill	*4.8*	*3.6*	*1.19*	*1.28*	*0.96 to 1.69*	*0.09*	*4.4*	*4.2*	0.12	–	–	–
*IUCD*	*3.2*	*3.1*	*0.15*	*1.07*	*0.78 to 1.48*	*0.67*	*3.8*	*3.1*	0.72	*1.20*	*0.40 to 3.63*	*0.74*
*Injectable method*	*1.8*	*1.6*	*0.16*	*1.11*	*0.70 to 1.78*	*0.65*	*3.2*	*1.5*	1.77	*1.27*	*0.46 to 3.48*	*0.64*
*Emergency contraceptive pill*	*0.34*	*0.53*	(*0.19*)	*0.61*	*0.26 to 1.41*	*0.25*	*0.0*	*0.5*	(0.49)	–	–	–
Sterilised (male or female) since birth of child	12.9	14.9	(2.00)	0.85	0.74 *to* 0.97	0.016	11.9	14.1	(2.20)	0.56	0.37 to 0.85	0.006
Current use of family planning	69.1	69.7	(0.62)	0.98	0.95 to 1.02	0.41	70.8	69.2	1.60	0.93	0.79 to 1.09	0.39
Ever used 1+ contraception methods since birth of child	60.3	58.4	1.85	1.03	0.97 to 1.09	0.41	64.9	58.7	6.20	1.19	0.84 to 1.68	0.33
Unmet need for family planning												
Women who could not get a method due to no stock	6.2	6.0	0.19	1.05	0.83 to 1.32	0.68	4.1	6.4	(2.32)	1.54	0.12 to 19.20	0.74
Breast feeding												
Immediate breast feeding	68.3	71.7	(3.45)	0.96	0.92 to 1.00	0.027	67.7	70.6	(2.90)	0.91	0.85 to 0.99	0.025
Immediate breast feeding: normal delivery	72.1	75.1	(3.02)	0.96	0.93 to 1.00	0.052	71.3	99.4	(28.10)	0.93	0.85 to 1.01	0.10
Ever breastfed	99.3	99.6	(0.34)	1.00	0.99 to 1.00	0.13	99.4	99.4	–	0.99	0.97 to 1.00	0.13
Never fed child <6 months janam ghutti, water, honey, animal milk, formula milk, jaifal or tea	11.3	10.6	0.71	1.07	0.91 to 1.26	0.40	14.0	10.5	3.50	1.21	0.72 to 2.04	0.48
Exclusive breast feeding*	10.8	10.4	0.36	1.04	0.88 to 1.23	0.64	13.4	10.1	3.30	1.10	0.67 to 1.81	0.71
Gave breastmilk in the previous 24 hours	95.6	96.1	(0.50)	1.00	0.98 to 1.01	0.47	95.6	95.8	(0.20)	0.98	0.92 to 1.04	0.47
Complementary feeding												
Child fed non-breastmilk before the child was 6 months of age	10.3	10.6	(0.25)	0.99	0.83 to 1.17	0.87	9.0	10.7	(1.68)	0.97	0.50 to 1.87	0.92
Introduced children to thicker foods at the age of 6 months	72.3	71.7	0.65	1.01	0.97 to 1.05	0.66	74.7	71.5	3.20	1.03	0.90 to 1.17	0.67
Infant feeding												
Gave child solid or semisolid food yesterday	98.9	98.9	0.05	1.00	0.99 to 1.01	0.68	98.8	98.9	(0.10)	1.01	0.98 to 1.04	0.68
Not yet giving child solid or semisolid food at time of interview	0.8	0.6	0.23	1.48	0.69 to 3.17	0.31	0.2	0.8	(0.54)	NA	NA	NA
Added oil/ghee to child’s food yesterday	42.9	41.8	1.07	1.04	0.97 to 1.11	0.28	44.9	41.9	3.00	1.13	0.90 to 1.41	0.30
Feeding frequency†	59.2	58.2	1.00	1.01	0.96 to 1.06	0.72	59.3	58.7	0.60	1.04	0.82 to 1.31	0.74
Minimum meal frequency‡	58.6	57.6	0.99	1.01	0.96 to 1.06	0.68	58.7	58.0	0.70	1.05	0.83 to 1.33	0.70
Dietary diversity*§	49.7	49.3	0.42	1.02	0.96 to 1.08	0.54	52.6	49.1	3.50	1.09	0.82 to 1.46	0.56
Child a minimum acceptable diet	31.9	29.9	1.98	1.07	0.98 to 1.17	0.13	34.8	30.4	4.40	1.31	0.88 to 1.95	0.18
Fed dal ka pani (contraindicated)	32.9	32.0	0.94	1.04	0.95 to 1.13	0.37	33.5	32.3	1.20	1.16	0.83 to 1.60	0.39
Iron folate												
Gave child iron syrup yesterday	3.5	3.3	0.28	1.08	0.79 to 1.47	0.65	3.4	3.4	(0.06)	1.38	0.37 to 5.14	0.63
Gave child iron rich diet yesterday	86.5	87.1	(0.56)	1.00	0.98 to 1.02	0.83	86.8	86.8	–	0.99	0.89 to 1.10	0.82

*Reported exclusive breast feeding in the first 6 months and have never fed their children janam ghutti, water, honey, animal milk, formula milk, jaifal or tea in the first 6 months of life.

†Fed child five times or more in the last 24 hours.

‡Child received solid, semisolid or soft foods the minimum number of times or more acording to Kilkari during the previous day.

§Child fed at least 1 food from 4 or more of the food groups.

CATE, compliance adjusted treatment effects; CF, complementary feeding; Diff, difference; FLHW, frontline health worker; FP, family planning; Int., intervention; IUCD, intrauterine contraceptive device; RR, relative risk.

### Secondary outcome: modern reversible contraceptive methods

**Figure 5:**
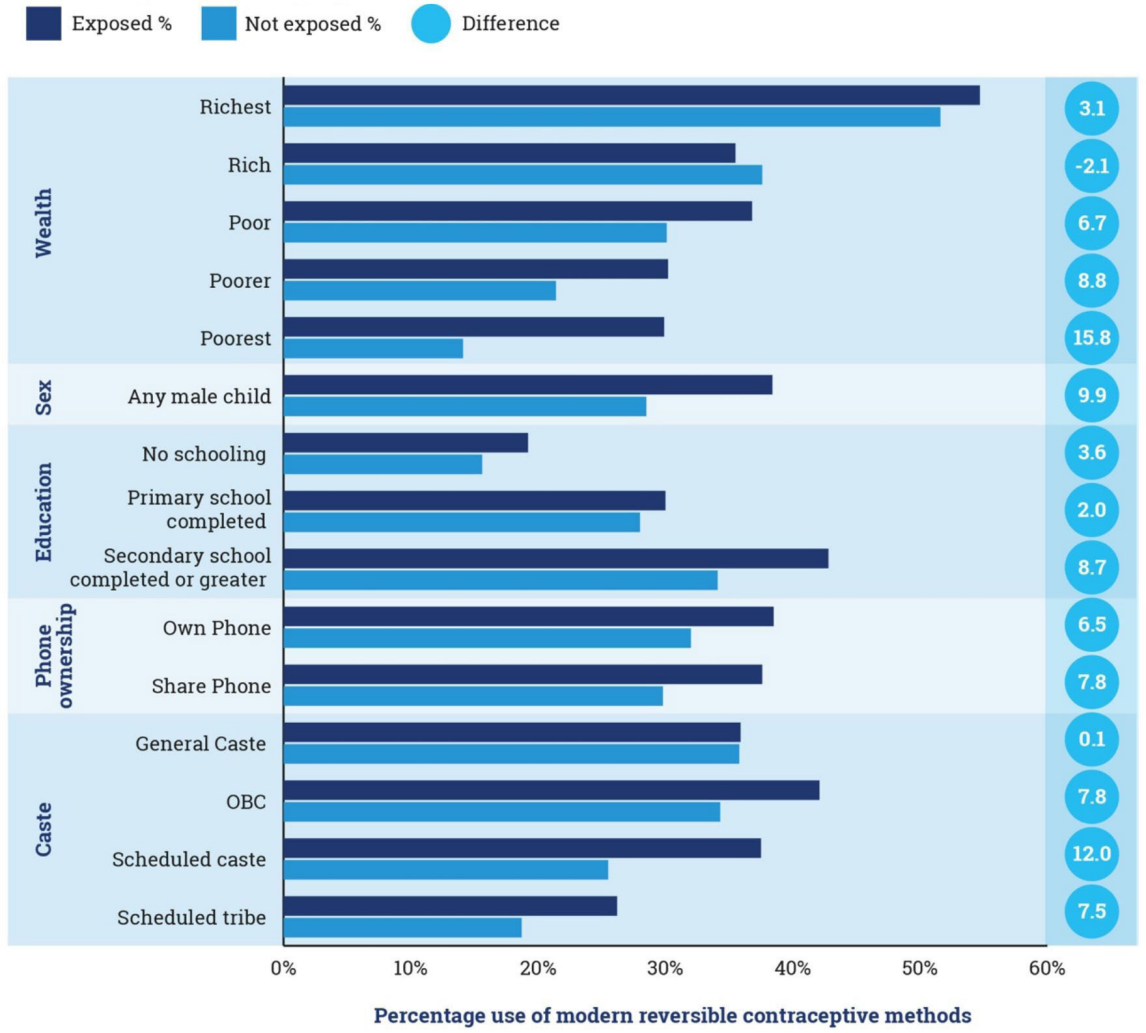
Proportion of those exposed versus not exposed using reversible modern contraceptive methods by sociodemographic characteristics. OBC, other backward castes.

Kilkari was associated with significantly higher reported use of modern reversible contraceptive methods (condoms, oral contraceptive pills, emergency contraceptive pills, injectables, IUCD) using both ITT (RR: 1.12, 95% CI 1.03 to 1.21, p=0.007) and IV (RR: 1.72, 95% CI 1.06 to 2.77, p=0.028) analyses, corresponding to an absolute difference of 3.7% across study arms and 7.4% higher among exposed versus not exposed groups (table 2). Differences in the use of reversible methods were driven by significantly higher use of condoms (ITT, RR: 1.12, 95% CI 1.01 to 1.23, p=0.024) and a marginally significant increase in the use of oral contraceptive pills (ITT, RR: 1.28, 95% CI 0.96 to 1.69, p=0.088). Subgroup analyses suggest that absolute differences in reversible method use between those exposed to Kilkari and those not exposed were highest for those with any male child (9.9% difference), in the lowest three socioeconomic strata (difference of 15.8% difference in the poorest, 8.8% in poorer and 6.7% in poor), and in scheduled castes (12.0% difference) (figure 5, online supplemental table 2A–C).

In addition to differences in the use of modern reversible contraceptive methods, the proportion of men or women sterilised since the birth of the child was 2.0% lower in the intervention arm (12.9%) than reported in the control arm (14.9%). Findings were significant across study arms (ITT, RR: 0.85, 95% CI 0.74 to 0.97, p=0.016) and among those exposed to Kilkari versus those not exposed (IV, RR: 0.56, 95% CI 0.37 to 0.85, p=0.006).

### Effects on other RMNCH practices

Table 3 presents impact findings for a range of other RMNCH practices, including facility delivery, essential newborn care, WASH and childhood immunisations. Findings suggest that the proportion of children immunised at 10 weeks was 2.8% higher in the intervention arm as compared with the control arm (ITT, RR: 1.04, 95% CI 1.00 to 1.07, p=0.048). Significant differences were not observed for other RMNCH practices assessed. Subgroup analysis found that differences in the proportion of children fully immunised at 12 months were higher among women exposed to Kilkari who had no schooling (difference of 15.2% across exposed vs not exposed groups) or primary school completed (difference of 9.9%) as compared with those with secondary school education or higher (difference of 1.4%) (online supplemental tables 2A–C). A similar trend was observed for immunisations at birth, 10 weeks, and 14 weeks.

**Table 3:** Delivery, essential newborn care, WASH and immunisation practices in four districts of Madhya Pradesh

Description	Women’s endline survey (n=4423)											
	Prevalence across study arms			Intention to treat			Prevalence across exposed versus not exposed groups			Instrumental variable		
	Int.	Control	Diff.	RR	95% CI	P value	Exposed	Not exposed	Diff.	CATE	95% CI	P value
Facility delivery												
Gave birth in a facility	94.4	93.4	0.94	1.01	1.00 to 1.03	0.17	96.5	93.6	2.9	1.05	0.98 to 1.13	0.17
Gave birth with a skilled attendant	95.0	94.4	0.66	1.01	0.99 to 1.02	0.30	98.3	94.3	4.0	1.04	0.96 to 1.12	0.31
Stayed in the facility for 2 days after giving birth	86.8	85.6	1.21	1.02	0.99 to 1.04	0.19	87.1	86.1	1.0	1.19	0.95 to 1.48	0.12
Essential newborn care (children n=4460)												
Hypothermia prevention												
Wrapped child after birth	93.7	94.5	(0.76)	1.00	1.00 to 1.01	0.43	99.3	98.9	0.3	1.01	0.99 to 1.02	0.43
Waited 2 days or more till bathing their child	80.7	81.7	(1.01)	0.99	0.96 to 1.02	0.41	83.3	80.6	2.7	0.97	0.91 to 1.04	0.40
Umbilical cord care: nothing on child’s umbilical cord	15.9	15.8	0.18	1.02	0.89 to 1.17	0.76	14.7	16.2	−1.5	1.06	0.73 to 1.53	0.78
Colostrum: fed their child colostrum	84.6	84.2	0.42	1.01	0.98 to 1.03	0.56	86.4	83.7	2.7	1.02	0.96 to 1.07	0.57
WASH												
Washed hands before feeding child last time	83.0	81.2	1.81	1.03	1.00 to 1.05	0.079	85.9	81.6	4.3	1.11	0.98 to 1.25	0.089
Phone numbers												
Kept phone numbers handy for emergencies	98.6	98.1	0.48	1.01	1.00 to 1.01	0.21	98.7	98.3	0.4	1.03	0.98 to 1.08	0.24
Kept FLHW number handy in case of emergency	92.4	92.0	0.47	1.00	0.99 to 1.02	0.62	94.8	91.9	2.9	1.02	0.94 to 1.11	0.60
Kept government ambulance number handy in case of emergency	61.6	60.5	1.09	1.03	0.98 to 1.08	0.30	67.9	60.4	7.5	1.15	0.87 to 1.52	0.33
Immunisations												
Child fully immunised at 12 months	41.7	40.5	1.22	1.02	0.95 to 1.10	0.53	44.7	40.6	4.2	1.09	0.84 to 1.41	0.54
Birth immunisations	79.0	78.4	0.61	1.01	0.98 to 1.04	0.70	81.0	78.3	2.7	1.02	0.92 to 1.13	0.70
6 weeks: OPV1/IPV1 and Penta 1	89.5	89.4	0.08	1.00	0.98 to 1.02	0.88	89.6	89.4	0.2	1.00	0.95 to 1.05	0.88
10 weeks: OPV2/IPV2 and Penta 2	74.8	72.0	2.81	1.04	1.00 to 1.07	0.048	75.4	73.0	2.4	1.10	1.00 to 1.22	0.057
14 weeks: OPV3/IPV3 and Penta 3	72.1	69.9	2.13	1.03	0.99 to 1.07	0.17	73.4	70.6	2.8	1.09	0.96 to 1.24	0.18
9 months: measles	81.5	83.0	(1.47)	0.98	0.96 to 1.01	0.24	84.6	82.0	2.7	0.92	0.81 to 1.05	0.22
Vitamin A: 1 or more	78.9	79.0	(0.07)	1.00	0.97 to 1.03	1.00	80.5	78.7	1.7	1.11	0.89 to 1.39	0.37
Timeliness of immunisations among those who had a vaccination card (n=3799)												
Birth: 0–2 weeks	75.9	77.2	(1.34)	0.98	0.95 to 1.02	0.30	77.8	76.2	1.6	1.01	0.87 to 1.16	0.93
6 weeks: 6–10 weeks	52.9	54.7	(1.79)	0.96	0.91 to 1.02	0.16	54.7	53.4	1.2	0.95	0.82 to 1.10	0.49
10 weeks: 10–14 weeks	27.8	28.6	(0.73)	0.96	0.87 to 1.06	0.47	29.2	27.9	1.3	0.97	0.75 to 1.25	0.80
14 weeks: 14–18 weeks	16.7	15.7	0.96	1.03	0.90 to 1.19	0.66	20.0	15.5	4.5	1.18	0.77 to 1.83	0.45
9 months: 39 weeks	40.0	42.0	(2.01)	0.96	0.89 to 1.03	0.23	43.4	40.7	2.7	0.87	0.62 to 1.21	0.41

CATE, compliance adjusted treatment effects; Diff, difference; FLHW, frontline health worker; Int., intervention; IPV, inactivated polio vaccine; OPV, oral polio vaccine; Penta, pentavalent; RR, relative risk; WASH, water sanitation hygiene.

### Effects on knowledge, decision-making, discussion, demand, supply

Tables 4–6 and online supplemental tables 3A, B and 4A, B present data on differences in women and men’s knowledge, decision-making, reported discussion, demand for and receipt of health information for RMNCH outcomes assessed. ITT findings suggest that the proportion of women (RR: 1.04, 95% CI 1.01 to 1.07, p<0.001) and men (RR: 1.05, 95% CI 1.00 to 1.09, p=0.032) who know to ask a health provider questions about family planning use was higher among those in the intervention arm compared with those in the control arm (table 2). This corresponds to a 3.4% and 3.0% difference across study arms for women and men, respectively. Similarly, both ITT (RR: 1.05, 95% CI 1.00 to 1.11, p=0.032) and IV (RR: 1.24, 95% CI: 1.01 to 1.53, p=0.044) findings suggest that women subscribed to Kilkari were more likely to know that they could obtain a pregnancy test from Accredited Social Health Activities, the Government of India’s community health worker cadre (table 5). Among a range of outcomes assessed on decision-making, discussion, demand and receipt of information, Kilkari was associated with an increased likelihood of women’s involvement in the decision-making on when to start giving their children complementary food (ITT RR: 1.03, 95% CI 1.00 to 1.06, p=0.041; IV RR: 1.11, 95% CI 1.00 to 1.24, p=0.042) (table 3). This corresponds to a 2.8% difference across study arms for women. Significant differences were not observed for other intermediate outcomes assessed.

**Table 4:** Women’s involvement in decision-making, discussion and demand for information in four districts of Madhya Pradesh

Description	Women’s endline survey (n=4423)											
	Prevalence across study arms			Intention to treat			Prevalence across exposed versus not exposed groups			Instrumental variable		
	Int.	Control	Diff.	RR	95% CI	P value	Exposed	Not exposed	Diff.	CATE	95% CI	P value
Decision-making												
Women involved in family planning or birth spacing decision-making	87.9	87.4	0.50	1.00	0.98 to 1.03	0.76	88.6	87.5	1.11	1.02	0.92 to 1.13	0.76
Women involved in the decision-making on when to start complementary feeding	84.3	81.6	2.75	1.03	1.00 to 1.06	0.041	84.7	82.8	1.94	1.11	1.00 to 1.24	0.042
Women involved in the decision about what complementary foods to give child	89.7	88.0	1.75	1.02	1.00 to 1.04	0.13	92.0	88.5	3.50	1.08	0.98 to 1.20	0.13
Discussion in last month												
Talked to family about family planning	49.8	49.1	0.73	1.01	0.95 to 1.07	0.74	50.8	49.3	1.50	1.05	0.80 to 1.39	0.72
Discussed family planning with FLHW	13.2	13.5	(0.30)	0.97	0.84 to 1.13	0.70	13.4	13.3	0.13	0.87	0.46 to 1.64	0.66
Talked to FLHW about complementary feeding	47.3	45.3	2.03	1.05	0.98 to 1.12	0.17	50.4	45.8	4.57	1.23	0.89 to 1.69	0.21
Discussed infant feeding with FLHW	19.4	21.3	(1.90)	0.90	0.80 to 1.01	0.085	19.8	20.4	−0.63	0.69	0.47 to 0.99	0.045
Spoke to FLHW about issues other than a child’s illness, feeding	3.9	3.9	0.01	0.99	0.73 to 1.33	0.94	2.7	4.0	−1.27	0.87	0.08 to 9.08	0.91
Discussed child’s illness with FLHW	17.5	18.5	(0.97)	0.95	0.83 to 1.07	0.38	16.4	18.2	−1.80	0.80	0.51 to 1.25	0.32
Demand for health information												
Asked an FLHW about family planning since birth of child	25.1	25.0	0.05	1.00	0.90 to 1.10	0.94	22.6	25.4	−2.76	0.99	0.59 to 1.66	0.96
Asked a health worker about family planning since birth of child	28.7	28.6	0.15	1.00	0.91 to 1.10	0.98	27.0	28.9	−1.88	1.01	0.64 to 1.61	0.96
Ever asked for advice on child’s immunisations	37.0	38.0	(0.91)	0.98	0.91 to 1.06	0.61	39.8	37.0	2.74	0.94	0.75 to 1.18	0.60
Ever asked FLHW for advice on child’s immunisations	32.1	33.0	(0.89)	0.98	0.90 to 1.06	0.60	34.4	32.2	2.19	0.94	0.73 to 1.20	0.60
Asked health worker for advice on child immunisation	32.7	33.7	(1.01)	0.98	0.90 to 1.06	0.56	34.8	32.9	1.95	0.93	0.73 to 1.19	0.56
Asked for advice on breast feeding	23.0	25.2	(2.19)	0.92	0.83 to 1.02	0.12	22.5	24.4	−1.88	0.76	0.56 to 1.04	0.088
Asked for advice from health worker on breast feeding	12.9	14.0	(1.04)	0.93	0.80 to 1.08	0.33	12.0	13.7	−1.64	0.77	0.48 to 1.24	0.28
Asked FLHW for advice on breast feeding	11.6	12.2	(0.57)	0.95	0.81 to 1.12	0.57	10.7	12.1	−1.33	0.84	0.49 to 1.45	0.54
Receipt (supply) of health information, services												
Told about family planning by a health worker since birth of child	49.9	50.8	(0.83)	0.98	0.92 to 1.04	0.46	49.2	50.5	−1.28	0.91	0.71 to 1.17	0.45
Ever received advice on child’s immunisations from FLHW	87.6	87.8	(0.18)	1.00	0.98 to 1.02	0.87	86.9	87.8	−0.87	0.99	0.93 to 1.07	0.86
Received advice on child immunisation from a health worker	88.5	88.9	(0.45)	1.00	0.97 to 1.02	0.66	87.9	88.8	−0.90	0.98	0.92 to 1.05	0.64
FLHW encourage/escorted women for child’s immunisation or health check	33.0	34.2	(1.15)	0.96	0.88 to 1.04	0.34	29.9	33.9	−3.99	0.77	0.48 to 1.24	0.28
Received an FLHW visit in the first 2 months postpartum	60.0	59.6	0.36	1.00	0.95 to 1.05	0.91	60.0	59.7	0.29	0.99	0.91 to 1.09	0.91
Received the all eligible FLHW visits in the first 2 months	1.1	1.0	0.04	1.04	0.58 to 1.86	0.90	1.60	0.83	0.77	1.01	0.47 to 2.20	0.97
Received advice on breast feeding	78.3	78.0	0.32	1.01	0.98 to 1.04	0.70	79.2	78.0	1.20	1.02	0.92 to 1.13	0.71
Received breast feeding advice from FLHW	61.9	60.2	1.68	1.03	0.98 to 1.08	0.23	62.0	61.0	1.01	1.11	0.93 to 1.31	0.24
FLHW encouraged/escorted them to a health facility in the last month	23.9	24.8	(0.85)	0.96	0.86 to 1.06	0.42	21.3	24.6	−3.32	0.76	0.43 to 1.35	0.35
FLHW gave them drugs or supplies in the last month	21.1	21.3	(0.19)	0.98	0.88 to 1.10	0.74	21.3	21.2	0.11	0.89	0.49 to 1.63	0.70

CATE, compliance adjusted treatment effects; CF, complementary feeding; Diff, difference; FLHW, frontline health worker; FP, family planning; Int., intervention; RR, relative risk.

**Table 5:** Family planning knowledge among women in four districts of Madhya Pradesh

Description	Women’s endline survey (n=4423)								
	Prevalence across study arms			Intention to treat			Instrumental variable		
	Int.	Control	Difference	RR	95% CI	P value	CATE	95% CI	P value
Family planning knowledge									
Safe methods for delaying pregnancy	93.0	92.7	0.35	1.00	0.99 to 1.02	0.70	1.02	0.94 to 1.10	0.71
One or more benefits of family planning	97.0	97.2	(0.22)	1.00	0.99 to 1.01	0.81	0.99	0.95 to 1.04	0.81
One or more benefits of small family	64.6	63.2	1.41	1.03	0.99 to 1.08	0.19	1.12	0.94 to 1.33	0.20
Source of pregnancy test									
FLHW	62.5	59.6	2.95	1.05	1.00 to 1.11	0.032	1.24	1.01 to 1.53	0.044
Health centre	61.9	63.2	(1.30)	0.98	0.94 to 1.03	0.43	0.93	0.80 to 1.10	0.40
Government provides free pregnancy tests	82.9	82.6	0.34	1.00	0.98 to 1.03	0.73	1.02	0.92 to 1.13	0.74
Birth spacing									
3 years gap is ideal	99.7	99.6	0.13	1.00	1.00 to 1.00	0.52	1.00	0.99 to 1.02	0.52
Know the benefits of a 3-year age gap	98.0	98.4	(0.36)	1.00	0.99 to 1.00	0.40	0.99	0.95 to 1.02	0.39
Postpartum pregnancy risk									
Woman can get pregnant before menses returns	52.7	53.4	(0.72)	0.98	0.93 to 1.04	0.54	0.96	0.84 to 1.09	0.53
Female sterilisation									
Ever heard of	99.8	99.7	0.12	1.00	1.00 to 1.00	0.38	1.01	0.99 to 1.02	0.38
Knowledge that services are free	97.4	96.6	0.82	1.01	1.00 to 1.02	0.14	1.03	0.99 to 1.08	0.15
Timing: can be done at time of birth	62.6	63.3	(0.64)	0.99	0.94 to 1.03	0.58	0.95	0.80 to 1.13	0.56
Male sterilisation									
Ever heard of	89.5	89.2	0.33	1.01	0.99 to 1.03	0.61	1.02	0.95 to 1.08	0.59
Knowledge that services are free	74.0	74.9	(0.93)	0.99	0.95 to 1.02	0.46	0.95	0.83 to 1.08	0.43
Causes men to become weak	73.7	73.1	0.60	1.01	0.98 to 1.05	0.52	1.05	0.90 to 1.24	0.52
Does not require incision or stitches	36.0	37.7	(1.73)	0.96	0.89 to 1.03	0.26	0.86	0.65 to 1.12	0.25
Postpartum intrauterine contraceptive device (PPIUCD)									
Ever heard of	82.7	82.9	(0.21)	1.00	0.97 to 1.03	0.98	1.00	0.93 to 1.08	0.98
Knowledge that services are free	69.0	68.9	0.11	1.00	0.96 to 1.04	0.91	1.01	0.90 to 1.12	0.93
Timing: can be done at time of birth	64.2	63.5	0.70	1.01	0.97 to 1.06	0.55	1.03	0.94 to 1.12	0.55
Awareness of other methods									
Medroxyprogesterone aceta/injectables	84.7	84.8	(0.09)	1.00	0.98 to 1.03	0.94	1.00	0.93 to 1.08	0.93
Oral contraceptive pills	89.0	87.2	1.80	1.02	1.00 to 1.04	0.063	1.11	0.99 to 1.24	0.075
Condoms	94.8	93.9	0.84	1.01	0.99 to 1.02	0.20	1.04	0.98 to 1.11	0.19
Emergency contraceptives	39.2	39.2	(0.03)	1.00	0.93 to 1.07	0.96	1.00	0.71 to 1.41	1.00
Health providers as source for information, methods									
Know to ask health provider questions about family planning use	83.8	80.4	3.36	1.04	1.01 to 1.07	0.0030	1.25	1.06 to 1.46	0.007
Keep FLHW number handy in case of emergency	95.1	94.7	0.36	1.00	0.99 to 1.02	0.66	1.02	0.95 to 1.09	0.66

CATE, compliance adjusted treatment effects; Diff, difference; FLHW, frontline health worker; FP, family planning; Int., intervention; OCPs, oral contraceptive pills; RR, relative risk.

**Table 6:** Family planning knowledge among men in four districts of Madhya Pradesh

Description	Men’s survey (n=3842)								
	Prevalence across study arms			Intention to treat			Instrumental variable		
	Int.	Control	Difference	RR	95% CI	P value	CATE	95% CI	P value
Family planning knowledge									
Safe methods for delaying pregnancy	96.3	96.7	(0.37)	1.00	0.98 to 1.01	0.55	0.98	0.92 to 1.04	0.55
One or more benefits of family planning	96.5	97.0	(0.45)	1.00	0.98 to 1.01	0.57	0.98	0.93 to 1.04	0.56
One or more benefits of small family	84.2	83.9	0.35	1.00	0.98 to 1.03	0.77	1.02	0.91 to 1.13	0.76
Source of pregnancy test									
FLHW	33.7	33.5	0.19	1.01	0.93 to 1.11	0.79	1.05	0.74 to 1.48	0.80
Health centre	59.1	59.3	(0.19)	1.00	0.94 to 1.05	0.89	0.99	0.80 to 1.21	0.90
Government provides free pregnancy tests	66.0	66.3	(0.37)	1.00	0.96 to 1.04	0.96	1.00	0.84 to 1.18	0.97
Birth spacing									
3 years gap is ideal	99.6	99.6	–	1.00	1.00 to 1.00	0.95	1.00	0.98 to 1.02	0.95
Know the benefits of a 3-year age gap	97.3	97.6	(0.32)	1.00	0.99 to 1.01	0.81	0.99	0.95 to 1.04	0.81
Postpartum pregnancy risk									
Woman can get pregnant before menses returns	46.4	44.9	1.54	1.04	0.97 to 1.12	0.24	1.10	0.94 to 1.30	0.24
Female sterilisation									
Ever heard of	99.4	99.6	(0.20)	1.00	0.99 to 1.00	0.48	0.99	0.97 to 1.01	0.48
Knowledge that services are free	96.2	95.8	0.41	1.01	0.99 to 1.02	0.44	1.02	0.97 to 1.08	0.43
Timing: can be done at time of birth	59.4	59.3	0.06	1.01	0.96 to 1.06	0.85	1.02	0.83 to 1.25	0.87
Male sterilisation									
Ever heard of	95.1	95.8	(0.67)	0.99	0.98 to 1.01	0.46	0.98	0.94 to 1.03	0.46
Knowledge that services are free	86.5	87.7	(1.15)	0.99	0.97 to 1.01	0.41	0.96	0.86 to 1.06	0.40
Causes men to become weak	59.4	59.1	0.33	1.00	0.95 to 1.05	0.91	0.99	0.78 to 1.26	0.92
Does not require incision or stitches	49.2	49.4	(0.12)	1.00	0.94 to 1.07	0.94	1.00	0.77 to 1.31	0.97
Postpartum intrauterine contraceptive device									
Ever heard of	58.8	56.9	1.90	1.05	1.00 to 1.10	0.070	1.16	0.99 to 1.35	0.070
Knowledge that services are free	43.8	43.6	0.27	1.02	0.95 to 1.09	0.58	1.06	0.88 to 1.28	0.55
Timing: can be done at time of birth	38.2	36.3	1.85	1.06	0.98 to 1.15	0.13	1.13	0.96 to 1.33	0.13
Awareness of other methods									
Medroxyprogesterone acetate/injectables	68.6	68.1	0.47	1.01	0.97 to 1.06	0.56	1.04	0.92 to 1.16	0.53
Oral contraceptive pills	87.2	85.7	1.42	1.02	0.99 to 1.04	0.13	1.10	0.97 to 1.25	0.13
Condoms	98.9	98.9	(0.02)	1.00	0.99 to 1.01	0.88	1.00	0.97 to 1.04	0.87
Emergency contraceptives	50.5	51.4	(0.89)	0.99	0.94 to 1.05	0.85	0.97	0.74 to 1.26	0.81
Health providers as source for information, methods									
Know to ask health provider questions about family planning use	70.6	67.6	2.98	1.05	1.00 to 1.09	0.032	1.27	1.00 to 1.62	0.047
Keep FLHW number handy in case of emergency	82.6	82.6	0.05	1.00	0.97 to 1.03	0.94	1.01	0.88 to 1.15	0.93

CATE, compliance adjusted treatment effects; CF, complementary feeding; Diff, difference; FLHW, frontline health worker; FP, family planning; Int., intervention; OCPs, oral contraceptive pills; RR, relative risk.

## Discussion

Study findings provide evidence on the exposure and impact of mHealth information messages across a range of RMNCH outcomes. Kilkari was not significantly associated with significant improvements in infant feeding practices, including the primary outcome of exclusive breast feeding. A significant increase was observed in the use of modern reversible contraceptive methods, particularly among those women exposed to Kilkari with any male child, in the poorest socioeconomic strata, and in disadvantaged ethnic groups. Increases in reversible method use were driven principally by increases in condom and oral contraceptive use, and occurred concurrently with a significant decrease in the proportion of men or women sterilised since the birth of the child. Increases in the proportion of children immunised at 10 weeks and the proportion of women involved in decision-making on when to introduce complementary foods were also observed. Significant improvements in other RMNCH outcomes were not observed. Findings suggest that an average of 65% of subscribers listened to more than 50% of the cumulative total of successful calls while 31% listened to more than 75% of the total content of calls. High listenership rates were stable over time; a likely indication that if calls reach the mobile device and are answered, subscribers tend to the listen to them. Analyses of the programme’s algorithm that attempts to call subscribers up to nine times to yield a successful call found that a greater number of call attempts are needed to reach the most marginalised.

Kilkari includes health information content across more than 11 health areas. The theory of change underpinning the evaluation is published elsewhere.^17^ Infant feeding outcomes were selected as the priority outcomes from the outset because they represent a sizeable share of message content (12%) and the practice of these behaviours is not supply side dependent, although immediate breast feeding can be. In the case of exclusive breast feeding, the low (58%) pretrial prevalence of this behaviour additionally left much potential for improvement.^19^ In practice, significant improvements were not observed in exclusive breast feeding. Qualitative research findings published elsewhere help to shed light on the factors underpinning these results.^23^ Kilkari messages that told women to give only breastmilk were interpreted to mean ‘give only breastmilk as the main source of nutrition’—since janam ghutti (a local term referring to homemade or indigenous herbal tonic), water and the provision of small quantities of sweets were not a source of nutrition, giving them to babies was not considered by caregivers to be incompatible with following the message to exclusively breastfeed.^23^ When Kilkari did specify *not* to give ghutti or other food items, women often did not seem to absorb this from the message.^23^ Broadly, women did not see valid reasons to stop giving substances such as ghutti which they thought helped to support the child’s health and in instances of illness could prevent worse illness. While quantitative survey findings did indicate a significant increase in the proportion of the mothers involved in decision-making over when to introduce complementary foods, mothers were also not the sole decision-makers and families too felt strongly that ghutti, water and other substances were helpful for babies. Women also reported appreciating and trusting the advice of elders who encouraged giving ghutti and other substances. Specific to the practice of breast feeding, most women in rural Madhya Pradesh reported having very limited support to exclusively breastfeed, including refrigeration, consistent electricity supply, pumping and bottle sterilisation, as well as support to solve physiological issues that may interfere with breast feeding. In sum, complex behaviours that have competing social norms and are currently seen to work quite well are challenging to change and receiving a small number of Kilkari voice calls advocating that one adopt a particular practice did not catalyse a change in behaviour.

From a measurement perspective, qualitative findings affirmed those from cognitive interviews done to support the design of survey tools.^24 25^ While the National Family Health Survey-4 findings from 2015 to 16 suggest that the reported practice of exclusive breast feeding was 58% in Madhya Pradesh, our findings suggest a much lower prevalence (intervention: 10.8%; control: 10.4%) when additional probing questions are incorporated in the survey tool. Discrepancies lie the wording of survey questions and indicator framing. Our estimates of exclusive breast feeding are based on two questions in contrast to other surveys: (1) women who reported practicing exclusive breast feeding in the first 6 months of their child’s life and (2) women who report never having fed their child janam ghutti, water, honey, animal milk, formula milk, jaifal or tea in the first 6 months of life. Across study arms, only 10.8% of women in the intervention arm and 10.4% of women in the control arm met this standard. Arguably this estimate is a more reliable indication of the true prevalence of exclusive breast feeding in this population.

In contrast to infant feeding, Kilkari had a significant impact on the secondary outcome of modern reversible contraceptive use. Family planning messages constituted the largest overall content area (18%) and were provided throughout the extended postpartum window. The impact on modern reversible contraceptive use was driven by increases in condom use and oral contraceptives, and as noted above, occurred concurrently with a decrease in sterilisation. A multitude of factors may explain these results. First, 49% of women in our sample reported sharing mobile phones and among women who did not own their own phones (25%), it was often their husbands who listened to Kilkari calls.^23^ Exposure to Kilkari calls among men may thus have catalysed some of the increase observed. Second, the study population was characterised by high economic migration among men; a factor which too may have influenced women’s decision-making on method preferences and the observed shift from permanent to reversible methods. Lastly, increases were significantly higher among subgroups including those with any male children. Given social norms around gender preferences for children, Kilkari may have served as a ‘tipping point’ to prompt behaviour change among those who had already had a male child. Indeed, qualitative findings suggest that men and women retained and appreciated Kilkari messages that aligned with their pre-existing worldviews, social norms and existing practices but overlooked or de-emphasised content that did not.^23^

### Comparison with findings elsewhere

Our study is the largest effectiveness trial conducted globally to date of a mHealth messaging programme operating at scale. Comparable RCTs of mHealth messaging programmes in other low resource settings were completed as part of pilot studies or smaller deployments in Africa.^8–11^ The changes in programme features and design which frequently occur as programmes transition from small scale pilots to large scale deployments can lead to a voltage drop in their effectiveness at scale and have implications for the generalisibiltiy of results.^2 15^ By starting with a programme already firmly established and scaled, we have sought to improve emerging evidence on the generalisability of such interventions. By recruiting study participants directly from their communities, we have sought to test the intervention under ‘imperfect’ real world conditions ensuring that findings are generalisable to and representative of the population at large with access to mobile phones. Further efforts to power the sampling frame to detect a 5% difference across study arms in the primary outcomes of reversible contraceptive method use and exclusive breast feeding sought to ensure that modest differences in outcomes would be detected. While challenges with exposure exceeded expectations (and are discussed below), subgroup analyses on key population subgroups were particularly critical to understanding the differential effect programme activities may have had. Elsewhere we continue to explore equity considerations in greater depth.^22^ Analyses of call data records made possible by MOHFW and with the support of technical partners including BBC Media Action, Beehyv and IMI Mobile were integral to our efforts to determine exposure to specific subsets of Kilkari messages by outcome and explore linkages between dosage of listening and reported health outcomes. Ours is the first evaluation of a mHealth messaging programme to make this link.

The RCT presented here is part of a large mixed methods evaluation of Kilkari.^17^ Findings add to the limited evidence available on the effectiveness of large-scale health information messaging programmes^4 7 12^ and smaller scale initiatives in Malawi^8^ and Zanzibar.^9–11^ However, comparisons with other programmes are challenging given the fundamental differences in programme components, implementation partners, study contexts, scale of implementation and evaluation design. Kilkari was designed as an integrated component of a suite of mHealth services that involved training of frontline health workers (Mobile Academy), equipping them with job aids (Mobile Kunji), and reinforcing health information provided to beneficiaries through contacts with the public health system (Kilkari).^2 16^ However, the programme was ultimately scaled as a standalone service with key features, including the timing of calls, modified based on what was feasible to implement at scale.^26^ By comparison, programmes in Bangladesh, Malawi, Zanzibar and elsewhere in India, all feature mHealth information messaging as part of a larger suite of services, often enabled by frontline health workers who provide face-to-face communication services, supported by an implementing non-government organisation with donor funding. The sole exception is South African National Department of Health’s MomConnect programme which does include a HelpDesk^27^ component but principally is centred around the delivery of 145 twice weekly text messages to new and expectant mothers.^28^

Globally, the future of mHealth messaging programmes remains uncertain. While several programmes have scaled to reach large numbers of subscribers,^4–7 28^ sustainability remains a continued challenge. While a number of factors underpin this, key drivers include dependency on donor funding, limited government capacity for oversight and stewardship, and lack of evidence on impact and value for money.^13^ The emergence of COVID-19 has further increased demands on already finite health resources. Despite the uncertainties, programmes like Kilkari, which have the infrastructure in place to send health information content out to millions of beneficiaries, offer much potential for delivering other kinds of health information, including information related to COVID-19. More broadly, our findings underscore the potential impact mHealth messaging programmes may have in providing women and their families with access to health information. By not restricting the framing of Kilkari messages to women, the programme may have tapped into a demand among men for health information. Further, efforts to ensure that Kilkari content was accessible regardless of subscribers’ education, caste or socioeconomic status, may underpin pro-poor findings observed for reversible contraceptive method use. Long-standing social norms, particularly those around son preference, are challenging to change. However, efforts to differentially target key population segments whom we know services are likely to impact the most may be one future avenue to explore.

### Limitations

Evaluation activities have several notable limitations. When compared with pregnant women at a population, our sample of women with access to a mobile phone during the day is likely more advantaged; a structural bias most mHealth initiatives face. However, when compared against women enrolled to the Kilkari programme elsewhere in India, the RCT sample may be different for two reasons. First, our RCT sample was identified via a household listing and screening survey where an estimated 50% of the pregnant women at a population level were eligible since they had access to a phone during the day. This exceeds the average population level coverage attained by Kilkari across 13 states in India (21%) and elsewhere in Madhya Pradesh (18%) which identifies subscribers passively through government tracking registries. Our RCT thus included a higher proportion of the population by including women who would not necessarily have been registered in the government’s tracking registry.^29^ The RCT faced challenges in ensuring continuous exposure to Kilkari messages among those in the intervention arm as a result of high rates of SIM change (44%), male population migration (potentially with their phones; 25% of men could not be interviewed as part of the endline survey), and increases in call non-delivery rates attributed to the bankruptcy of the telecommunications provider for the Kilkari programme, in mid-2018. All of these factors exceeded initial expectations and resulted in a loss of power due to lower compliance and programme exposure. A third limitation of the study lies in our reliance on self-reported outcomes for all outcomes except immunisations, many of which may be subject to social desirability bias. A fourth limitation lies in the information and recall biases associated with collecting data on practices occurring over a long window at a single time point (12 months postpartum). Additionally, we were not able to measure outcomes related to self-efficacy to practice RMNCH behaviours. Lastly, there may be unmeasured confounders but we assume the randomisation process distributed them evenly across the two arms.

## Conclusions

Study findings provide the most conclusive evidence to date on the effectiveness of a mHealth messaging initiative at scale in India and globally. The moderate impact observed on modern reversible contraceptive method use, particularly among the most marginalised, is promising while the lack of any measurable changes in infant feeding behaviours is disappointing. Further research is needed to understand whether the impact might be deepened through microtargeting-specific subsets of the beneficiary population with tailored programme content; by changing the number of messages and the duration of the service; and by complementing Kilkari with supplementary face to face communication to create an enabling environment more supportive of behaviour change.

10.1136/bmjgh-2022-008838.supp2 Supplementary data

## Data Availability

Data are available upon reasonable request. Anonymised data are available upon reasonable request from the study Principal Investigator Dr. Amnesty LeFevre aelefevre@gmail.com.
