# Supplementary material for: The impact of a direct to beneficiary mobile communication program on reproductive and child health outcomes: a randomised controlled trial in India
Source: BMJ Glob Health. 2022 Jul 14;6(Suppl 5):e008838. doi: 10.1136/bmjgh-2022-008838 (PMC9288869; doi:10.1136/bmjgh-2022-008838)
Supplement: Supplementary data [file bmjgh-2022-008838supp001.pdf]

Supplementary Figure 1. Odds of listening to 50% or more of cumulative content

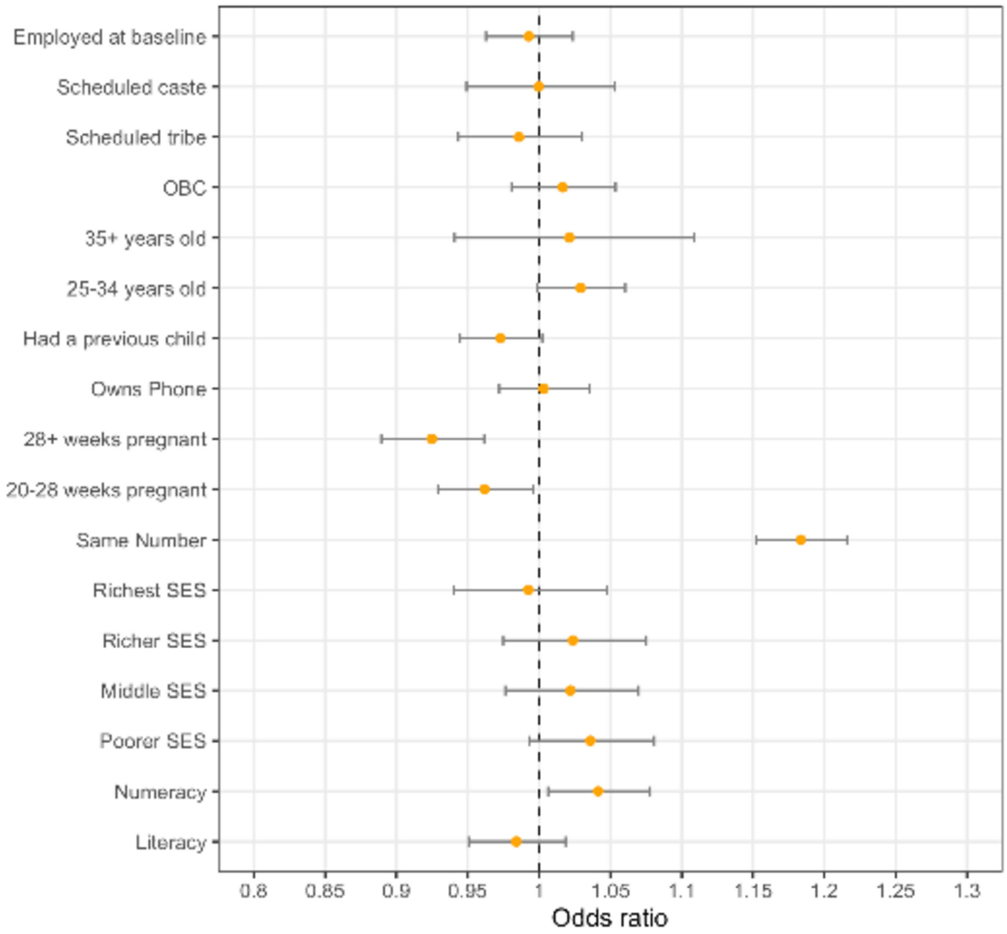

Supplementary Table 1. Kilkari messages on reversible contraceptives

Delivery week	Message Number	Message Title	Message	Duration in seconds			
				Topic	Message	Closing	**Total**
Pregnancy month 8, week 4	20	Family planning - PPIUCD	Like many families you might be worried about unwanted pregnancies. A woman can get pregnant again right after delivery even if she is breastfeeding. The woman can get an IUCD inserted for free at a government health facility which can protect you from unwanted pregnancies for a long time. It can be inserted within 48 hours after delivery while you are at the hospital so you wont have to make another trip. And remember you can have it removed whenever you want to have a baby. It's very simple and free. Contact your ASHA/ANM immediately for more information.	20	72	16	108
Pregnancy month 9, week 4	24	PPFP - New	The time right after delivery is a good time to adopt a method of family planning. This will give you more time to take care of the baby. After the delivery, before the women leaves the health facility, she can has two family planning options: first, PPIUCD insertion or second, female sterilization. The PPIUCD can be inserted within 48 hours after delivery which is effective up to 5-10 years and can get it removed when she wants to have another child. Postpartum sterilization can be performed within 7 days of delivery which is a permanent method and can be adopted by the woman when her family is complete and she doesn't want any more children. These services are available free of cost at all government health facilities. For more information contact your ASHA or ANM. Remember it is your right to ask your ASHA, ANM or doctor about family planning options, to be treated with respect, and it is your right to avail these services in a clean and safe government health facility.	15	71	15	86
Baby month 2, week 1	29	Introducing MPA injection for FP	There are several methods of birth control mthods available now. Explains about the MPA injection method of family planning, MPA injection, safe and effective - save time, money and better future. One injection prevents pregnancy for 3 months and no effect on breastfeeding. With Doctor's advance any woman can take MCP injection 6 weeks post delivery. MPA card that reminds that tracks your next MPA injection date. If any problem with the MCP injection talk to your ASHA or health center. Times are changing, spacing between kids will make small family happy family. Speak to your ASHA/ANM/Doctor so you can make an informed decision about which family planning method to use.	18	90	15	108
Baby month 3, week 2	34	IUCD	Are you worried about getting pregnant again? The IUCD is a simple and effective solution to your worries. There are two kinds of IUCDs - one that lasts for 5 years and one that lasts for 10 years. It can be inserted for free at a health facility by a trained doctor or nurse 6 weeks post delivery. And remember you can have it removed whenever you want to have a baby. The IUCD is inserted for free. Talk to your ASHA/ANM about choosing an IUCD today.	17	62	13	79
Baby month 11, week1	65	OCPs - new *Chaaya* pill	Worried about unwanted pregnancy? Govt of India has launched a new tablet, called *Chaaya.* You can take this pill even if you are breastfeeding or thereafter. There is no negative impact on your body with this pill. You can start taking these pills if you are not pregnant. You can get these new pills *Chaaya* or other OCPs from health centers or from your ASHA. It might affect your mestrual cycle in the beginning but don't worry it means that the pill is having the desired effect. You just need to ensure that they are taking the right dose of *Chaaya* at the right time and in the right way. If you face any problem after taking the pill talk to your ASHA.	17	75	15	92
Baby month 11, week 2	66	Condoms	If you're looking for a quick and handy family planning method then consider the condom. The condom is an easy to use contraceptive method for men which protects you from unplanned pregnancy as well as sexually transmitted infections and HIV. But, it is very important for you to remember to use a condom everytime you have sex in order for it to be effective. Make it a habit and you can stop worrying about unwanted pregnancies. Condoms are available at any government hospital and with ASHAs. Contact your ASHA or ANM for more information.	17	52	15	69

Baby month 11, week 4	68	ECPs	If you are in a situation where you had unprotected sex and are now worried that you may be pregnant, then you have an option - the emergency contraceptive pill. The Emergency Contraceptive Pill helps prevent pregnancy when taken within 72 hours of unprotected sex, but it is ineffective after 72 hours. But remember, the EC pill is not a regular contraceptive method and should be used only in case of an emergency. Contact your ASHA or ANM for more information.	16	59	15	75
			**Subtotal**	**120**	**481**	**104**	**617**

Supplementary Table 2a. Sub-group analyses of family planning and infant feeding practices amongst those exposed to Kilkari as compared to those not exposed in four districts of Madhya Pradesh

	Reversible methods				Sterilized				Immediate breastfeeding (among normal births)				Exclusive breastfeeding				Minimum acceptable diet			
	Exposed		Not exposed		Exposed		Not exposed		Exposed		Not exposed		Exposed		Not exposed		Exposed		Not exposed	
	n	%	n	%	n	%	n	%	n	%	n	%	n	%	n	%	n	%	n	%
**Wealth**																				
Richest	70	54.7	406	51.6	13	10.8	92	11.6	130	72.22	371	73.2	23	15.5	114	14.7	58	41.1	329	42.1
Rich	39	35.5	301	37.6	13	9.8	116	14.9	128	65.64	436	73.2	19	13.2	84	10.8	54	41.5	273	34.4
Poor	39	36.8	234	30.1	13	11.2	108	14.1	139	69.15	448	73.4	19	13.8	71	9.5	33	29.5	239	30.9
Poorer	32	30.2	163	21.4	16	14.3	126	16.7	162	71.37	447	73.4	19	13.2	57	7.8	36	30.8	191	25.3
Poorest	23	29.9	108	14.1	13	14.0	102	13.6	149	78.42	497	77.7	12	10.6	54	7.3	23	26.4	144	18.7
**Any male child**	138	38.4	733	28.5	66	16.7	519	20.5	470	70.25	1472	74.4	67	14.6	251	10.1	133	33.5	739	28.9
**Education**																				
No schooling	10	19.2	66	15.6	8	13.1	87	21.1	76	69.72	240	71.0	5	7.4	46	11.3	11	17.7	78	18.8
Primary school completed	24	30.0	180	28.0	9	10.6	116	18.2	116	64.8	351	70.2	24	21.8	77	12.4	27	33.3	170	26.2
Secondary school or greater	169	42.8	966	34.1	51	11.9	341	12.2	516	73.19	1608	75.7	63	12.4	257	9.4	166	37.4	928	33.0
**Phone access**																				
Own Phone	157	38.5	940	32.0	48	11.2	400	13.7	547	72.84	1636	74.0	76	14.1	292	10.3	151	34.5	922	31.4
Share Phone	92	37.6	547	29.8	37	13.0	275	15.3	349	70.79	1052	74.4	39	12.2	166	9.3	97	33.9	506	27.9
**Caste**																				
General	47	35.9	308	35.8	13	9.6	93	10.9	143	70.79	435	72.9	17	10.6	73	8.7	47	34.8	296	34.3
OBC	112	42.1	623	34.3	34	11.5	257	14.4	345	69.7	999	72.8	56	15.6	205	11.8	116	38.2	592	33.0
Scheduled caste	33	37.5	198	25.5	11	12.0	122	15.8	127	72.57	500	77.6	12	11.2	81	10.6	27	27.0	197	25.5
Scheduled tribe	11	26.2	83	18.7	10	20.4	72	16.5	93	76.86	265	75.9	7	11.3	21	4.9	14	29.2	91	20.5
**Husband available**	181	42.5	1060	34.7	59	12.9	450	14.9	550	70.15	1698	73.7	82	14.7	330	11.2	166	34.9	984	32.4

Supplementary Table 2b. Sub-group analyses of immunization practices overall, at birth and six weeks amongst those exposed to Kilkari as compared to those not exposed in four districts of Madhya Pradesh

	Fully immunized				Immunizations at birth				Immunizations at 6 weeks			
	Exposed		Not exposed		Exposed		Not exposed		Exposed		Not exposed	
	n	%	n	%	n	%	n	%	n	%	n	%
**Wealth**												
Richest	69	49.6	380	48.5	118	81.9	617	79.3	172	92.5	683	92.8
Rich	66	54.6	351	43.7	135	87.1	632	82.2	179	93.7	655	89.4
Poor	61	44.9	318	42.4	110	82.7	593	78.8	188	89.1	605	89.6
Poorer	42	35.6	277	36.8	105	77.2	572	77.8	168	88.0	600	88.2
Poorest	36	36.4	234	30.9	94	74.6	533	72.9	140	84.3	600	86.8
**Any male child**	193	46.3	1000	39.4	378	80.4	1916	77.1	564	89.7	2082	89.5
**Education**												
No schooling	31	48.4	137	33.3	53	77.9	306	75.0	92	91.1	331	88.3
Primary school completed	39	48.2	248	38.3	85	82.5	474	75.7	152	89.9	508	90.7
Secondary school completed or greater	204	43.6	1175	42.2	424	81.1	2167	79.3	603	89.3	2304	89.3
**Phone access**												
Own Phone	213	44.9	1183	40.8	429	79.4	2219	78.3	645	89.6	2370	89.3
Share Phone	134	46.2	718	39.7	277	81.7	1379	78.4	403	90.6	1476	89.3
**Caste**												
General Caste	65	45.1	358	42.0	122	81.3	647	76.4	193	90.2	706	90.2
OBC	133	44.8	759	42.1	287	82.5	1402	80.1	409	90.1	1484	90.2
Scheduled caste	45	40.5	298	39.1	89	76.7	589	77.8	153	86.0	616	88.6
Scheduled tribe	31	50.8	145	33.7	64	80.0	309	75.2	92	92.9	337	86.0
**Husband available**	233	46.9	1278	42.4	453	81.8	2348	79.3	687	89.3	2473	90.1

Supplementary Table 2c. Sub-group analyses of immunization practices at 10 weeks, 14 weeks and 9 months amongst those exposed to Kilkari as compared to those not exposed in four districts of Madhya Pradesh

	Immunizations at 10 weeks				Immunizations at 14 weeks				Immunizations at 9 months			
	Exposed		Not exposed		Exposed		Not exposed		Exposed		Not exposed	
	n	%	n	%	n	%	n	%	n	%	n	%
**Wealth**												
Richest	162	83.9	578	79.3	128	81.5	606	79.2	98	94.2	722	88.3
Rich	143	76.1	561	76.2	126	84.0	570	73.6	77	84.6	711	85.4
Poor	125	72.3	519	72.8	103	67.3	510	69.6	84	82.4	666	85.0
Poorer	121	75.2	504	71.0	98	70.0	505	69.1	74	81.3	613	78.6
Poorest	103	67.8	461	65.4	76	61.8	447	60.9	52	77.6	570	72.2
**Any male child**	428	76.0	1738	72.6	352	72.7	1741	70.4	258	83.8	2150	81.2
**Education**												
No schooling	63	65.6	241	63.4	57	74.0	235	58.9	34	77.3	317	73.4
Primary school completed	104	77.6	424	71.3	75	71.4	428	68.6	57	80.3	523	79.5
Secondary school or greater	487	76.5	1958	74.8	399	73.8	1975	72.8	294	86.5	2442	83.8
**Phone access**												
Own Phone	484	74.7	2006	73.6	424	75.2	2002	71.3	271	84.2	2524	82.7
Share Phone	321	77.2	1195	71.1	234	70.9	1237	70.0	203	87.1	1505	80.7
**Caste**												
General caste	152	77.2	591	73.9	131	78.0	600	72.4	89	88.1	748	83.5
Other backward caste	318	75.9	1261	75.1	256	73.1	1281	73.2	193	85.0	1550	82.8
Scheduled caste	123	78.3	505	70.5	99	73.3	490	66.4	65	78.3	648	82.0
Scheduled tribe	61	64.9	266	67.0	45	64.3	267	63.4	38	86.4	336	75.2
**Husband available**	539	76.5	2113	75.2	431	74.4	2141	73.0	311	84.7	2630	83.6

Supplementary Table 3a. Infant and young child feeding knowledge among women in four districts of Madhya Pradesh

	Women's endline survey (n = 4,423)										
	Prevalence across study arms			Intention to Treat				Instrumental Variable			
Description	Int.	Comp.	Difference	RRS	95% CI		p	CATE	95% CI		p
Infant and young child feeding											
1+ signs that a child is not getting enough food to eat	98.4	98.5	(0.04)	1.00	0.99	1.01	0.93	1.00	0.97	1.03	0.93
Colostrum											
Know colostrum should be fed	86.4	86.5	(0.08)	1.00	0.98	1.02	1.00	1.00	0.95	1.05	0.99
1+ benefits of colostrum	85.6	84.7	0.90	1.01	0.99	1.04	0.29	1.03	0.98	1.08	0.29
Incorrectly think Colostum is NOT beneficial and/or think colostrum is bad for the baby	13.8	13.0	0.82	1.06	0.91	1.23	0.47	1.13	0.79	1.63	0.50
Breastfeeding, transition to complementary foods											
Timing: breastfeeding should start immediately or 1 hour after birth	87.2	88.2	(1.05)	0.99	0.97	1.01	0.33	0.98	0.93	1.02	0.32
1+ benefits of exclusive breastfeeding	98.6	98.3	0.34	1.00	1.00	1.01	0.35	1.01	0.99	1.04	0.33
Frequency: newborn should be breastfed 8+ times a day or on demand	86.2	87.2	(1.01)	0.99	0.96	1.01	0.22	0.97	0.91	1.02	0.22
1+ difficulty breastfeeding	97.5	97.2	0.37	1.00	0.99	1.01	0.40	1.01	0.99	1.04	0.41
Duration	-	-									
Incorrectly think the transition to complementary foods should start before 6 months	4.6	5.2	(0.57)	0.89	0.69	1.15	0.37	0.75	0.41	1.39	0.36
Introduce foods, water and liquids other than breastmilk after 6 months of age	92.0	91.4	0.62	1.01	0.99	1.02	0.44	1.02	0.96	1.09	0.45
Incorrectly reported that mother's should introduce foods, water and liquids other than breastmilk after 7 months of age	3.2	3.3	(0.14)	0.93	0.68	1.27	0.66	0.73	0.11	4.62	0.73
Give children thick consistency food at the age of 6 months	99.8	99.9	(0.07)	1.00	1.00	1.00	0.61	1.00	0.99	1.01	0.61
Contraindicated foods											
Babies should NOT be fed dal ka pani	2.5	2.2	0.25	1.13	0.77	1.65	0.54	1.41	0.36	5.52	0.62
Incorrectly think babies over 6 months should be fed dal ka pani	58.5	58.3	0.15	1.01	0.96	1.06	0.74	1.03	0.86	1.23	0.73
Food groups											
Children over 6 months of age should be given oil/ghee	11.1	9.4	1.71	1.19	1.00	1.42	0.05	2.02	0.79	5.16	0.14
Know at least 1 food from 4+ of the food groups for a child at 6 months of age	45.8	43.9	1.88	1.05	0.98	1.12	0.14	1.30	0.89	1.89	0.17
Child over 6 months should be fed food 3 or 4 times a day	59.5	60.1	(0.61)	1.00	0.95	1.05	0.89	0.99	0.86	1.13	0.88
Frequency: child at 1 year of age should be fed 5 times a day	25.7	27.9	(2.25)	0.92	0.83	1.01	0.09	0.70	0.49	1.00	0.05
Minimum acceptable diet: child 6 months of age or older should receive a minimum acceptable diet	29.1	27.8	1.31	1.06	0.96	1.16	0.24	1.29	0.82	2.03	0.27
Anemia											
1+ signs of anaemia	96.0	96.8	(0.86)	0.99	0.98	1.00	0.17	0.96	0.92	1.01	0.16
Anemia can be detected through a blood test at health facility/ AWW	31.1	32.0	(0.89)	0.96	0.88	1.05	0.35	0.84	0.60	1.18	0.31
Know what foods can be given to a child to improve iron in the blood	92.7	93.1	(0.38)	1.00	0.98	1.01	0.67	0.98	0.92	1.06	0.67

Know that Iron/ IFA syrup can be given to a child above 6 months to improve iron level in blood,	30.4	28.5	1.85	1.08	0.98	1.18	0.11	1.24	0.93	1.66	0.14
Iron rich foods to give a child	89.3	87.9	1.46	1.02	1.00	1.04	0.10	1.09	0.98	1.21	0.12
Other child health											
Give breastmilk if a child gets diarrhea	10.6	11.7	(1.11)	0.94	0.80	1.10	0.44	0.81	0.50	1.29	0.37
Additional feeding during child illness	23.2	24.4	(1.11)	0.96	0.87	1.07	0.50	0.87	0.60	1.27	0.48

Supplementary Table 3b. Infant and young child feeding knowledge among men in four districts of Madhya Pradesh

	Men's survey (n=3,842)										
	Prevalence across study arms			Intention to Treat				Instrumental Variable			
Description	Int.	Comp.	Difference	RRS	95% CI		p	CATE	95% CI		p
Infant and young child feeding											
1+ signs that a child is not getting enough food to eat	98.1	98.6	(0.49)	1.00	0.99	1.00	0.28	0.98	0.94	1.02	0.28
<u>Colostrum</u>											
Know colostrum should be fed	78.6	79.3	(0.72)	0.99	0.96	1.03	0.70	0.99	0.93	1.05	0.70
1+ benefits of colostrum	77.2	77.9	(0.76)	0.99	0.96	1.03	0.72	0.99	0.92	1.06	0.72
Incorrectly think Colostum is NOT beneficial and/or think colostrum is bad for the baby	21.4	18.6	2.83	1.14	1.00	1.29	0.05	1.36	0.98	1.89	0.07
Breastfeeding, transition to complementary foods											
<u>Timing</u>: breastfeeding should start immediately or 1 hour after birth	65.5	66.3	(0.76)	0.99	0.95	1.04	0.76	0.98	0.89	1.08	0.75
1+ benefits of exclusive breastfeeding	97.7	98.8	(1.04)	0.99	0.98	1.00	0.03	0.97	0.94	1.00	0.02
<u>Frequency</u>: newborn should be breastfed 8+ times a day or on demand	68.6	70.0	(1.36)	0.98	0.94	1.02	0.42	0.96	0.87	1.06	0.40
1+ difficulty breastfeeding	89.5	88.9	0.53	1.01	0.99	1.03	0.47	1.02	0.96	1.08	0.46
<u>Duration</u>											
Incorrectly think the transition to complementary foods should start before 6 months	11.0	8.7	2.29	1.25	1.03	1.52	0.02	3.33	0.55	20.05	0.19
Introduce foods, water and liquids other than breastmilk after 6 months of age	78.3	79.8	(1.51)	0.99	0.96	1.02	0.41	0.95	0.85	1.07	0.42
Incorrectly reported that mother's should introduce foods, water and liquids other than breastmilk after 7 months of age	10.6	11.3	(0.71)	0.90	0.75	1.07	0.23	0.68	0.36	1.28	0.23
Give children thick consistency food at the age of 6 months	99.5	99.2	0.23	1.00	1.00	1.01	0.34	1.01	0.99	1.03	0.35
Contraindicated foods											
Babies should NOT be fed dal ka pani	4.0	2.8	1.21	1.42	1.02	1.97	0.04	NA	NA	NA	NA
Incorrectly think babies over 6 months should be fed dal ka pani	55.5	59.8	(4.28)	0.93	0.88	0.98	0.01	0.79	0.67	0.92	0.00
Food groups											
Children over 6 months of age should be given oil/ghee	2.7	2.3	0.34	1.16	0.78	1.71	0.47	1.41	0.54	3.71	0.48
Know at least 1 food from 4+ of the food groups for a child at 6 months of age	39.5	41.4	(1.90)	0.95	0.89	1.03	0.22	0.82	0.63	1.08	0.16
Child over 6 months should be fed food 3 or 4 times a day	59.3	57.6	1.72	1.03	0.98	1.09	0.23	1.11	0.93	1.33	0.25
Frequency: child at 1 year of age should be fed 5 times a day	23.5	24.7	(1.14)	0.96	0.86	1.07	0.44	0.82	0.54	1.27	0.37
Minimum acceptable diet: child 6 months of age or older should receive a minimum acceptable diet	23.0	24.5	(1.57)	0.94	0.84	1.05	0.27	0.80	0.56	1.14	0.23
Anemia											
1+ signs of anaemia	95.5	96.1	(0.56)	0.99	0.98	1.01	0.42	0.97	0.91	1.04	0.41

Anemia can be detected through a blood test at health facility/ AWW	15.1	15.5	(0.43)	0.96	0.82	1.12	0.63	0.86	0.47	1.55	0.61
Know what foods can be given to a child to improve iron in the blood	88.6	89.8	(1.18)	0.99	0.97	1.01	0.26	0.94	0.86	1.04	0.22
Know that Iron/ IFA syrup can be given to a child above 6 months to improve iron level in blood,	27.5	30.1	(2.68)	0.92	0.83	1.01	0.09	0.79	0.62	1.01	0.06
Iron rich foods to give a child	74.6	76.7	(2.11)	0.98	0.94	1.01	0.19	0.90	0.77	1.05	0.16
Other child health											
Give breastmilk if a child gets diarrhea	4.8	3.8	1.06	1.27	0.94	1.72	0.13	4.91	0.05	507	0.50
Additional feeding during child illness	9.7	10.0	(0.21)	1.00	0.82	1.21	1.00	0.99	0.47	2.09	0.98

Supplementary Table 4a. Delivery, postnatal care, and child health knowledge among women interviewed at endline in four districts of Madhya Pradesh

	Women's endline survey (n = 4,423)										
	Prevalence across study arms			Intention to Treat				Instrumental Variable			
Description	Int.	Comp.	Difference	RRS	95% CI		p	CATE	95% CI		p
Labor and delivery knowledge											
Know at least 1 of 3 consequences of preterm labor	94.4	94.0	0.36	1.00	0.99	1.02	0.70	1.02	0.92	1.12	0.71
Stay in the facility for 2 days after giving birth	95.8	95.8	0.02	1.00	0.99	1.01	0.88	1.01	0.92	1.10	0.88
Postnatal care											
Danger signs for newborn babies	97.5	97.6	(0.06)	1.00	0.99	1.01	0.85	1.00	0.98	1.02	0.85
Umbilical cord care											
Redness or oozing could mean poor healing	95.8	96.1	(0.26)	1.00	0.99	1.01	0.65	0.99	0.96	1.02	0.66
Put nothing on the cord	11.6	12.3	(0.72)	0.95	0.82	1.11	0.55	0.89	0.61	1.29	0.53
Hypothermia prevention											
Delayed bathing: wait 48 hours before bathing child	70.6	71.7	(1.02)	0.98	0.95	1.02	0.36	0.96	0.88	1.05	0.35
Wrap, cover head, cover feet, delay bathing after delivery	93.7	94.5	(0.76)	0.99	0.98	1.01	0.34	0.98	0.95	1.02	0.33
Child immunizations											
Know at least 1 disease vaccines protect against	83.4	84.7	(1.23)	0.99	0.96	1.01	0.29	0.95	0.87	1.04	0.27
Vaccination card awareness	1.4	1.2	0.15	1.12	0.67	1.86	0.67	1.89	0.14	26.38	0.63
Timing: know vaccination starts at birth	82.8	82.3	0.54	1.01	0.98	1.04	0.55	1.03	0.94	1.13	0.55
Vaccines available for											
Diarrhea	11.5	10.7	0.79	1.07	0.90	1.26	0.44	1.22	0.71	2.10	0.48
Measles	37.9	39.4	(1.55)	0.96	0.89	1.03	0.26	0.91	0.76	1.09	0.29
Polio	73.8	74.1	(0.28)	1.00	0.96	1.03	0.79	0.99	0.90	1.08	0.78
Vitamin A supplementation											
1+ benefit of giving child vitamin	71.8	70.7	1.10	1.02	0.99	1.06	0.19	1.09	0.95	1.25	0.20
Diarrhea											
Give ORS if a child gets diarrhea	45.4	44.8	0.63	1.01	0.95	1.08	0.67	1.07	0.84	1.35	0.60
Give zinc if a child gets diarrhea	13.7	13.3	0.41	1.05	0.90	1.21	0.54	1.15	0.74	1.80	0.53
Give antidiarrheals, antibiotics, or other	82.7	81.3	1.43	1.02	0.99	1.05	0.16	NA	NA	NA	NA
1+ times when to wash hands	99.9	99.9	0.01	1.00	1.00	1.00	0.81	1.00	0.99	1.01	0.81
Pneumonia											
1+ symptoms of a child having pneumonia	93.4	92.9	0.50	1.01	0.99	1.02	0.54	1.02	0.95	1.10	0.54
Early childhood development											

Four month old child should be able to join both hands together	11.4	9.7	1.72	1.19	1.00	1.41	0.06	2.14	0.71	6.46	0.18
Six month old child should be able to reach for an object and raise his head	32.3	29.6	2.71	1.09	1.00	1.20	0.05	1.47	0.94	2.29	0.09
1 year old child should be able to walk with external suppoert, hold objects in its hands, and throw or play with objects	40.2	38.7	1.51	1.03	0.96	1.11	0.44	1.19	0.76	1.87	0.44
1+ actions to take worried about child development	39.7	37.2	2.55	1.08	1.00	1.16	0.05	1.33	0.97	1.82	0.08
Contact ASHA/ANM if they are worried about their child's ECD	26.2	24.6	1.59	1.08	0.97	1.19	0.16	1.31	0.86	1.99	0.21
Entitlements											
Ever heard of RBSK	8.1	7.3	0.85	1.11	0.91	1.37	0.30	1.60	0.60	4.25	0.35
RBSK provideds free check-ups for children, tests physical development, and tests mental development	4.4	4.2	0.24	1.07	0.81	1.41	0.63	1.25	0.50	3.12	0.64
Services provided under RBSK	5.0	4.5	0.54	1.13	0.87	1.48	0.36	1.51	0.55	4.14	0.42
Mentioned RBSK to check child development	0.9	0.7	0.26	1.44	0.71	2.93	0.31	16.75	0.00	-	0.84
Know to ask ASHA / ANM / AWW about RBSK screening if child is late in learning	9.8	10.5	(0.68)	0.95	0.80	1.12	0.53	0.84	0.49	1.45	0.54
Ask ASHA/ANM/AWW about RBSK screening if they are worried about child's ECD	34.3	33.4	0.94	1.04	0.96	1.13	0.37	1.15	0.84	1.57	0.38
Health systems services											
Know to keep government ambulance number handy in case of emergency	71.7	71.2	0.43	1.01	0.97	1.05	0.65	1.05	0.85	1.28	0.66

Supplementary Table 4b. Delivery, postnatal care, and child health knowledge among men interviewed at endline in four districts of Madhya Pradesh

	Men's survey (n=3,842)										
	Prevalence across study arms			Intention to Treat				Instrumental Variable			
Description	Int.	Comp.	Difference	RRS	95% CI		p	CATE	95% CI		p
Labor and delivery knowledge											
Know at least 1 of 3 consequences of preterm labor	92.9	93.5	(0.61)	1.00	0.98	1.01	0.58	0.97	0.88	1.07	0.56
Stay in the facility for 2 days after giving birth	96.1	96.7	(0.62)	0.99	0.98	1.00	0.22	0.95	0.87	1.03	0.21
Postnatal care											
Danger signs for newborn babies	95.8	94.8	0.97	1.01	1.00	1.02	0.17	1.02	0.99	1.05	0.17
Umbilical cord care											
Redness or oozing could mean poor healing	83.7	84.6	(0.91)	0.99	0.96	1.02	0.50	0.98	0.92	1.04	0.50
Put nothing on the cord	1.8	1.8	0.00	0.99	0.62	1.58	0.97	0.96	0.28	3.34	0.95
Hypothermia prevention											
Delayed bathing: wait 48 hours before bathing child	63.0	64.7	(1.72)	0.98	0.93	1.02	0.34	0.94	0.84	1.06	0.33
Wrap, cover head, cover feet, delay bathing after delivery	97.2	97.3	(0.09)	1.00	0.99	1.01	0.94	1.00	0.97	1.03	0.94
Child immunizations											
Know at least 1 disease vaccines protect against	88.7	89.6	(0.92)	0.99	0.97	1.02	0.52	0.97	0.89	1.06	0.53
Vaccination card awareness	1.3	1.7	(0.38)	0.82	0.48	1.41	0.48	0.61	0.17	2.12	0.43
Timing: know vaccination starts at birth	53.5	51.8	1.72	1.04	0.98	1.10	0.19	1.15	0.94	1.42	0.18
Vaccines available for			0.00								
Diarrhea	18.8	17.2	1.60	1.09	0.95	1.25	0.21	1.34	0.80	2.24	0.27
Measles	34.9	35.9	(1.06)	0.98	0.90	1.07	0.64	0.96	0.78	1.17	0.67
Polio	86.2	86.9	(0.67)	1.00	0.97	1.02	0.72	0.99	0.92	1.06	0.73
Vitamin A supplementation											
1+ benefit of giving baby vitamin A	77.8	79.3	(1.45)	0.99	0.96	1.02	0.46	0.96	0.86	1.07	0.44
Diarrhea											
Give ORS if a child gets diarrhea	53.0	54.7	(1.64)	0.98	0.93	1.03	0.47	0.94	0.79	1.13	0.52
Give zinc if a child gets diarrhea	10.6	11.2	(0.60)	0.95	0.79	1.13	0.55	0.85	0.51	1.41	0.52
Give antidiarrheals, antibiotics, or other	77.5	79.4	(1.97)	0.98	0.94	1.01	0.15	NA	NA	NA	NA
1+ times when to wash hands	100.0	99.9	0.01	1.00	1.00	1.00	0.94	1.00	0.99	1.01	0.94
Pneumonia											
1+ symptoms of a child having pneumonia	93.1	92.6	0.52	1.01	0.99	1.02	0.52	1.03	0.95	1.11	0.51
Early childhood development											
Four month old child should be able to join both hands together	6.0	4.8	1.20	1.25	0.96	1.63	0.09	7.26	0.01	5,386	0.56

Six month old child should be able to reach for an object and raise his head	20.7	18.7	1.97	1.11	0.98	1.26	0.11	1.87	0.69	5.12	0.22
1 year old child should be able to walk with external suppoert, hold objects in its hands, and throw or play with objects	51.7	50.2	1.51	1.03	0.97	1.10	0.28	1.21	0.84	1.74	0.31
1+ actions to take worried about child development	34.5	32.8	1.69	1.05	0.96	1.15	0.31	1.18	0.85	1.64	0.32
Contact ASHA/ANM if they are worried about their child's ECD	27.1	23.4	3.72	1.15	1.03	1.29	0.01	1.71	1.03	2.84	0.04
Entitlements											
Ever heard of RBSK	14.4	13.8	0.59	1.09	0.94	1.27	0.24	1.35	0.72	2.53	0.36
RBSK provideds free check-ups for children, tests physical development, and tests mental development	7.6	6.9	0.77	1.15	0.92	1.44	0.22	1.62	0.68	3.88	0.28
Services provided under RBSK	7.9	7.1	0.74	1.15	0.92	1.43	0.22	1.61	0.67	3.91	0.29
Mentioned RBSK to check child development	2.8	2.1	0.70	1.37	0.92	2.04	0.12	4.61	0.10	215	0.44
Know to ask ASHA / ANM / AWW about RBSK screening if child is late in learning	10.7	9.0	1.71	1.19	0.98	1.45	0.09	1.99	0.72	5.53	0.19
Ask ASHA/ANM/AWW about RBSK screening if they are worried about child's ECD	30.8	27.6	3.15	1.11	1.00	1.23	0.04	1.48	0.97	2.25	0.07
Health systems services											
Know to keep government ambulance number handy in case of emergency	91.2	92.0	(0.83)	0.99	0.97	1.01	0.48	0.96	0.86	1.07	0.49
